# 7‐Ketodeoxycholic Acid Promotes Colonic Mucosal Healing by Inducing Calcium Release from Endoplasmic Reticulum via the TGR5‐IP3R Pathway

**DOI:** 10.1002/advs.202507953

**Published:** 2025-09-15

**Authors:** Jing Zhang, Feng Jiang, Wenxin Xia, Yilei Guo, Yanrong Zhu, Mianjiang Zhao, Lingzi Xiao, Zhifeng Wei, Yufeng Xia, Yue Dai

**Affiliations:** ^1^ Department of Pharmacognosy School of Traditional Chinese Pharmacy China Pharmaceutical University Nanjing 211198 P. R. China; ^2^ Department of Pharmacology of Chinese Materia Medica School of Traditional Chinese Pharmacy China Pharmaceutical University Nanjing 211198 P. R. China; ^3^ Affiliated Hospital of Nanjing University of Chinese Medicine Nanjing 210029 P. R. China

**Keywords:** 7‐ketodeoxycholic acid, IP3R, mucosal healing, TGR5, ulcerative colitis

## Abstract

Defective healing of injured mucosa is a hallmark of many pathological conditions, such as ulcerative colitis (UC). Wound healing is a pivotal process that is essential for the reconstruction of epithelial homeostasis following damage to mucous membrane. However, the endogenous metabolites capable of expediting intestinal mucosal healing remain largely undefined. The aim of this study is to identify a pro‐repair metabolite to accelerate colonic wound healing. The investigation reveals that the serum levels of 7‐ketodeoxycholic acid (7‐KDCA), deoxycholic acid (DCA), and lithocholic acid (LCA) are depleted in patients with UC and colitic mice relative to controls. Among the three bile acids, 7‐KDCA exhibits the most conspicuous, which is correlated with disease severity. 7‐KDCA treatment exerts the strongest promotion of mucosal healing in mice with dextran sulfate sodium‐induced mucosal damage or biopsy‐induced colonic wounding injury. Mechanistically, 7‐KDCA functions by driving intestinal epithelial cell migration through induction of calcium release from the endoplasmic reticulum via targeting the TGR5‐IP3R axis. Amidst the array of endogenous metabolites potentially active in progression of UC, 7‐KDCA stands out as the preeminent facilitator in the healing of colonic mucosa. This finding may hold clinical significance for treating mucosal defect‐related diseases, including UC.

## Graphical abstract

7‐KDCA functions by driving intestinal epithelial cell migration through induction of calcium release from the endoplasmic reticulum. The findings emphasize a previously unrecognized property of 7‐KDCA in regulating cell migration, a required process for effective epithelial restitution and repair that is particularly critical for patients with UC to promote healing of mucosal wounds and ulcerations during remission phases of disease.

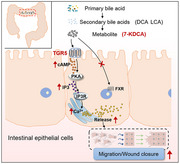

## 1 Introduction

Ulcerative colitis (UC) manifests as a recurring gastrointestinal disorder and is characterized by damage to the colonic mucosal epithelium. Clinical reports indicate that the initiation and continuation of mucosal healing in those with UC proves advantageous in diminishing the recurrence rate, hospitalization rate, surgical interventions needed, and the risk of colorectal cancer.^[^ ^1^, ^2^ ^]^ The layers of intestinal epithelial cells (IECs) act as a defense, shielding the body from environmental affronts. In the event that tissue integrity is compromised due to damage, IECs collectively migrate to enshroud the exposed region, thus sealing the breach.^[^ ^3^ ^]^ Swift healing of wounds is imperative for the preservation of intestinal barrier integrity. Disruption of this process results in an extended period of mucosa being exposed to luminal substances, eliciting recurring inflammatory responses.^[^ ^4^, ^5^ ^]^

Recently, the triggers and intermediaries of healing have emerged as areas of intense research interest. Several endogenous metabolites, such as bile acids, kynurenine, and indole‐3‐aldehyde have been shown to trigger mucosal healing through different mechanisms.^[^ ^6^, ^7^, ^8^, ^9^, ^10^ ^]^ Cholecystokinin can induce the discharge of bile acids into the intestinal lumen of mice and promote IEC regeneration by increasing cell proliferation.^[^ ^6^ ^]^ Ursodeoxycholic acid enhances wound healing by driving IEC migration.^[^ ^7^ ^]^ Deoxycholic acid (DCA) promotes crypt regeneration by inhibiting the production of prostaglandin E2, thereby accelerating wound repair.^[^ ^8^ ^]^ Kynurenine expedites IL‐10‐dependent wound closure of intestinal epithelium through the activation of aryl hydrocarbon receptor.^[^ ^9^ ^]^ Indole‐3‐aldehyde prompts LPLs to secret IL‐22, subsequently activating STAT3 pathway to facilitate the restoration of impaired intestinal mucosa.^[^ ^10^ ^]^ Of the many metabolites identified in patients and animal models of UC, only the above‐mentioned metabolites have been proven to exert a promotive influence on intestinal mucosal healing. Therefore, we conducted a more extensive investigation to ascertain if other metabolites possess a superior capacity to enhance the healing of the intestinal mucosa.

Our findings in the present study indicated a significant decrease of circulating 7‐ketodeoxycholic acid (7‐KDCA), DCA, and lithocholic acid (LCA) levels in patients with UC and colitic mice. Of these three bile acids, 7‐KDCA showed most potent effect in promoting mucosal healing in two mouse models of colon injury. 7‐KDCA functioned by promoting IEC migration through stimulation of calcium release from the endoplasmic reticulum via Takeda G protein‐coupled receptor 5 (TGR5). As a naturally occurring TGR5 agonist, 7‐KDCA is a promising candidate drug in the realm of preventing and treating mucosal defect‐related diseases, including UC.

## 2 Results

### 2.1 The Levels of 7‐KDCA, DCA, and LCA are Decreased in Patients with UC and Colitic Mice

To explore the plausible involvement of endogenous metabolites in the onset of UC, we conducted a comparative analysis of serum metabolic profiles from 32 patients with UC and 14 healthy controls (Figure S1A, Supporting Information). The OPLS‐DA diagram showed notable differences in serum metabolites between HC and patients with UC (Figure S1B, Supporting Information). Compared with those in the serum of HC, the levels of 10 endogenous metabolites were upregulated in the serum of patients with UC, while those of 7 endogenous metabolites were downregulated (Figure S1C, Supporting Information, online Table S1, Supporting Information). The levels of 7‐KDCA, linoleic acid, and indole‐3‐carboxaldehyde were remarkably decreased in patients with UC compared with HC (Figure S1C, Supporting Information, online Table S1, Supporting Information). These observations align with the existing literature, indicating that patients with UC and colitic mice have lower levels of linoleic acid and indole‐3‐carboxaldehyde.^[^ ^11^, ^12^, ^13^ ^]^ To our knowledge, this is the first report indicating a notable reduction of serum 7‐KDCA level in patients with UC.

**Figure 1:**
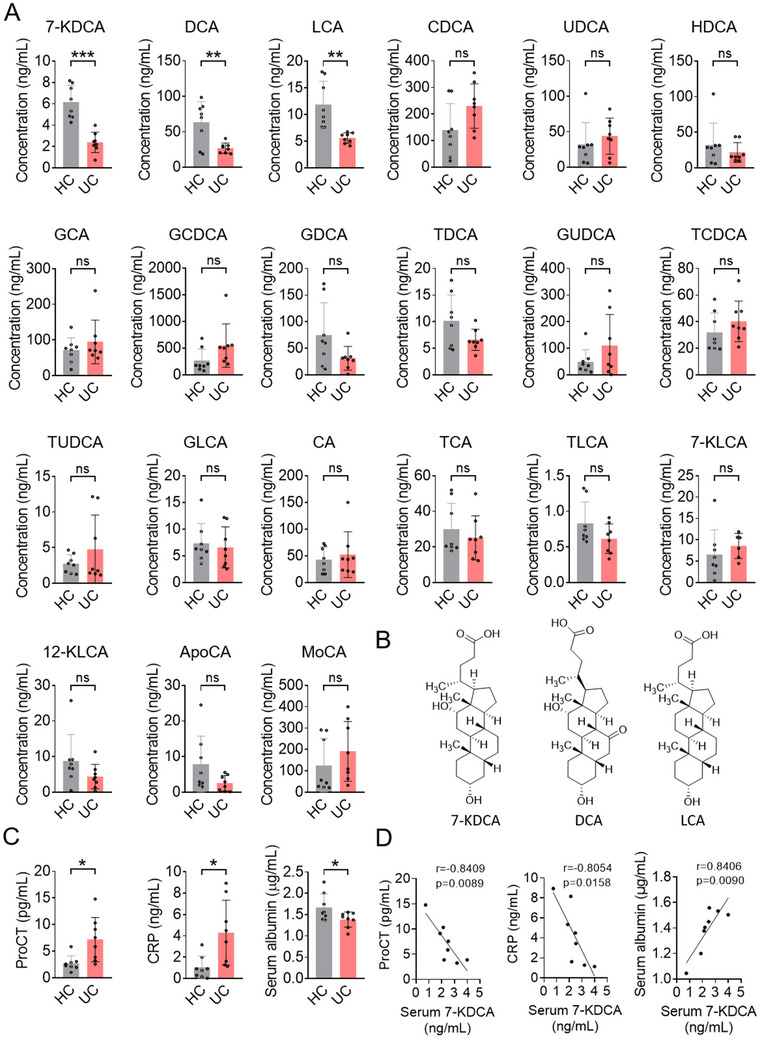
Levels of 7‐KDCA, DCA, and LCA are decreased in UC patients. A) Serum bile acids concentration in HC (n=8) and UC patients (n=8). B) Chemical structures of 7‐KDCA, DCA, and LCA. C) ProCT, CRP, and serum albumin levels in HC (n=8) and UC patients (n=8). D) The correlation between 7‐KDCA levels and ProCT, CRP, as well as serum albumin levels in the serum of UC patients (n=8). Data points represent individual subjects. ^*^ *p* < 0.05, ^**^ *p* < 0.01, ^***^ *p* < 0.001 vs. HC group; ns, no significant difference. 7‐KDCA, 7‐ketodeoxycholic acid; DCA, deoxycholic acid; LCA, lithocholic acid; UC, ulcerative colitis; HC, healthy controls; ProCT, procalcitonin; CRP, C‐reactive protein.

To authenticate the alterations in bile acids in UC, we recruited an additional cohort consisting of eight UC patients, alongside eight age‐ and sex‐matched HC, from the Jiangsu Province Hospital of Chinese Medicine to serve as the validation set. We employed a targeted metabolomics investigation utilizing LC‐MS to quantify different bile acids. The most notable discrepancy in the bile acid profiles was a substantial reduction in the levels 7‐KDCA, DCA, and LCA in patients with UC (**Figure** **1**A,B). No substantial variance was observed in the other bile acids of patients with UC (Figure 1A). Subsequently, we assessed the severity of UC by examining various laboratory markers, including procalcitonin (ProCT), C‐reactive protein (CRP), and albumin levels (Figure 1C).^[^ ^14^ ^]^ The level of 7‐KDCA exhibited an inverse correlation with the circulating levels of ProCT and CRP (Figure 1D). Further correlation analysis also substantiated a positive correlation between 7‐KDCA and the serum concentrations of albumin (Figure 1D). These data suggest a correlation between the serum concentration of 7‐KDCA and the severity of human UC.

**Figure 2:**
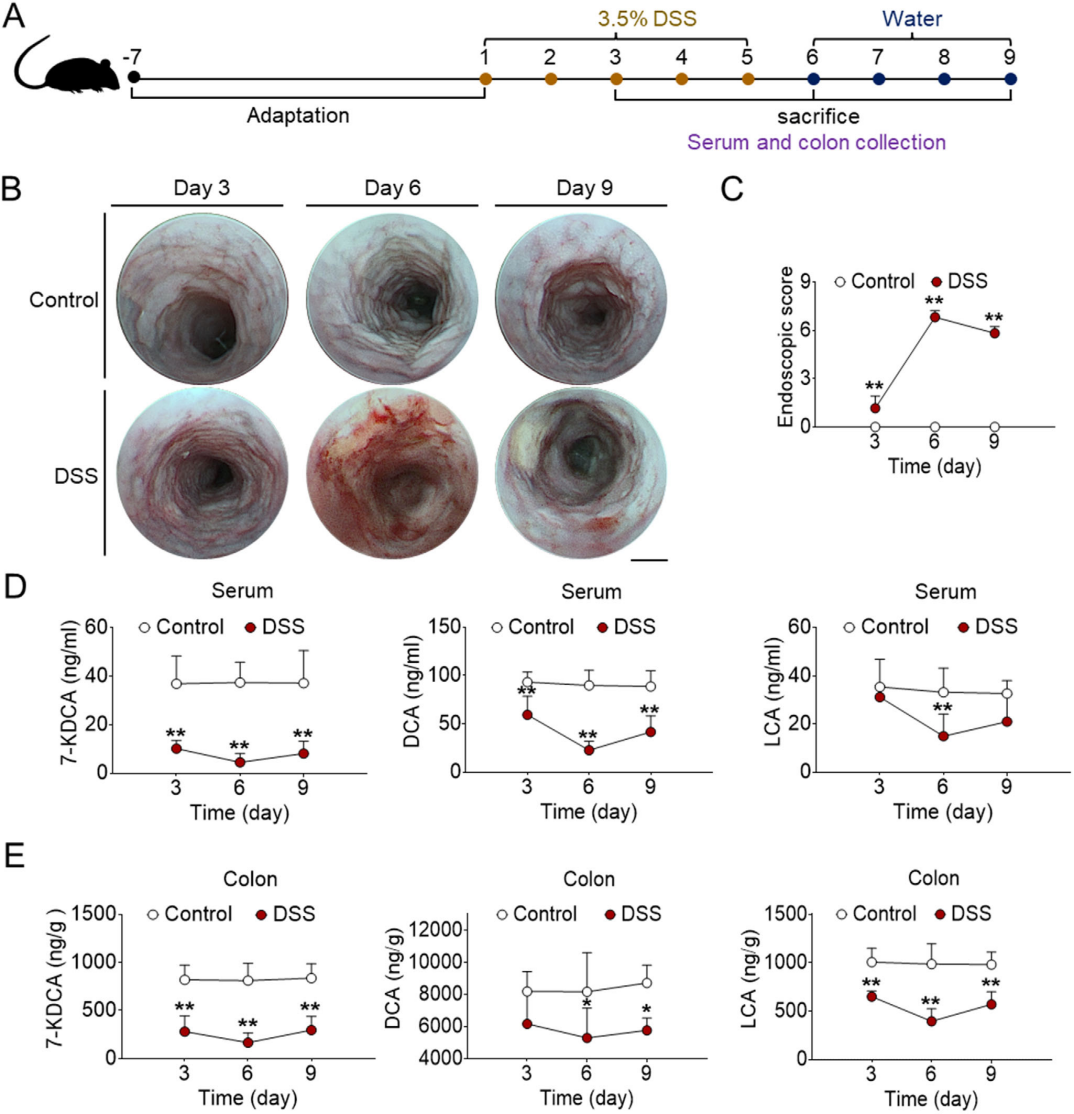
The levels of 7‐KDCA, DCA, and LCA are decreased in experimental colitic mice. Mice were treated either with or without 3.5% DSS for 5 days, succeeded by 4 days of water exposure. Colon and serum samples were harvested on days 3, 6, and 9 after DSS consumption. A) Illustrative diagram delineating the colitis model induced by DSS. B) Representative images displaying regions characterized by overt bleeding and edema. C) Endoscopic score. Levels of 7‐KDCA, DCA, and LCA in serum D) and colon E). Data represent mean ± SD (n = 6). ^*^ *p* < 0.05, ^**^ *p* < 0.01 vs. control group. 7‐KDCA, 7‐ketodeoxycholic acid; DCA, deoxycholic acid; LCA, lithocholic acid; DSS, dextran sulfate sodium.

In addition, a comparable observation was noted in mice subjected to dextran sulfate sodium (DSS)‐induced colitis, a frequently employed chemically induced experimental model for colitis. The progression of DSS‐induced colitis was separated into the induction phase, acute phase, and recuperation phase based on the extent of disease damage.^[^ ^15^ ^]^ The induction period was considered to begin on the third day after DSS exposure. The sixth day and ninth days after DSS exposure were delineated as the acute stage and recuperation stage, respectively. Colon and serum samples were harvested on days 3, 6, and 9 after DSS exposure, with the sample collection in the control group following the same schedule (**Figure** **2**A). Within the trio of phases, the colonic impairment in mice with colitis reached its peak during the acute stage, as evidenced by alterations in body weight change, disease activity index (DAI) score, myeloperoxidase (MPO) activity, intestinal permeability, and colon length (Figure S2, Supporting Information). To visualize the degree of healing in the interior mouse colon tissue, endoscopic examination was conducted. Results showed progressive worsening of disease in DSS mice, which exhibited persistent bleeding and edema, along with abundant and large mucosal ulcerations, peaking on day 6 (Figure 2B,C).

Consistent with the findings in humans, we observed a decrease of circulating 7‐KDCA, DCA, and LCA levels in colitic mice. Dynamic analysis of 7‐KDCA, DCA, and LCA during and post‐DSS treatment revealed that a progressive decline in the levels of these three bile acids began as early as day 3 following the initiation of DSS challenge, reaching a nadir on day 6; they returned to levels closely resembling the norm after DSS removal (Figure 2D,E), suggesting that the three bile acids levels were associated with colonic pathological changes in colitic mice. The level of 7‐KDCA in serum is positively correlated with that in the colon (Figure S3, Supporting Information), indicating a decrease of 7‐KDCA in intestinal synthesis leading to a reduction in circulation level. In addition, the most notable discrepancy in the bile acid profile of colon tissues was a substantial reduction in the levels 7‐KDCA, DCA, LCA and 7‐ketolithocholic acid in colitic mice (Figure S4, Supporting Information). No substantial variance was observed in the other bile acids of colitis group (Figure S4, Supporting Information). Importantly, the levels of 7‐KDCA, DCA and LCA in colonic tissue were extremely abundant, significantly higher than those in the serum (Figure S4, Supporting Information). Interestingly, while the serum concentration of 7‐KDCA is significantly lower than that of primary bile acids such as CDCA and GCDCA, its concentration in colonic tissue is notably higher than that of these primary bile acids.

### 2.2 Oral Administration of 7‐KDCA Promotes Colonic Mucosal Repair In Vivo

**Figure 3:**
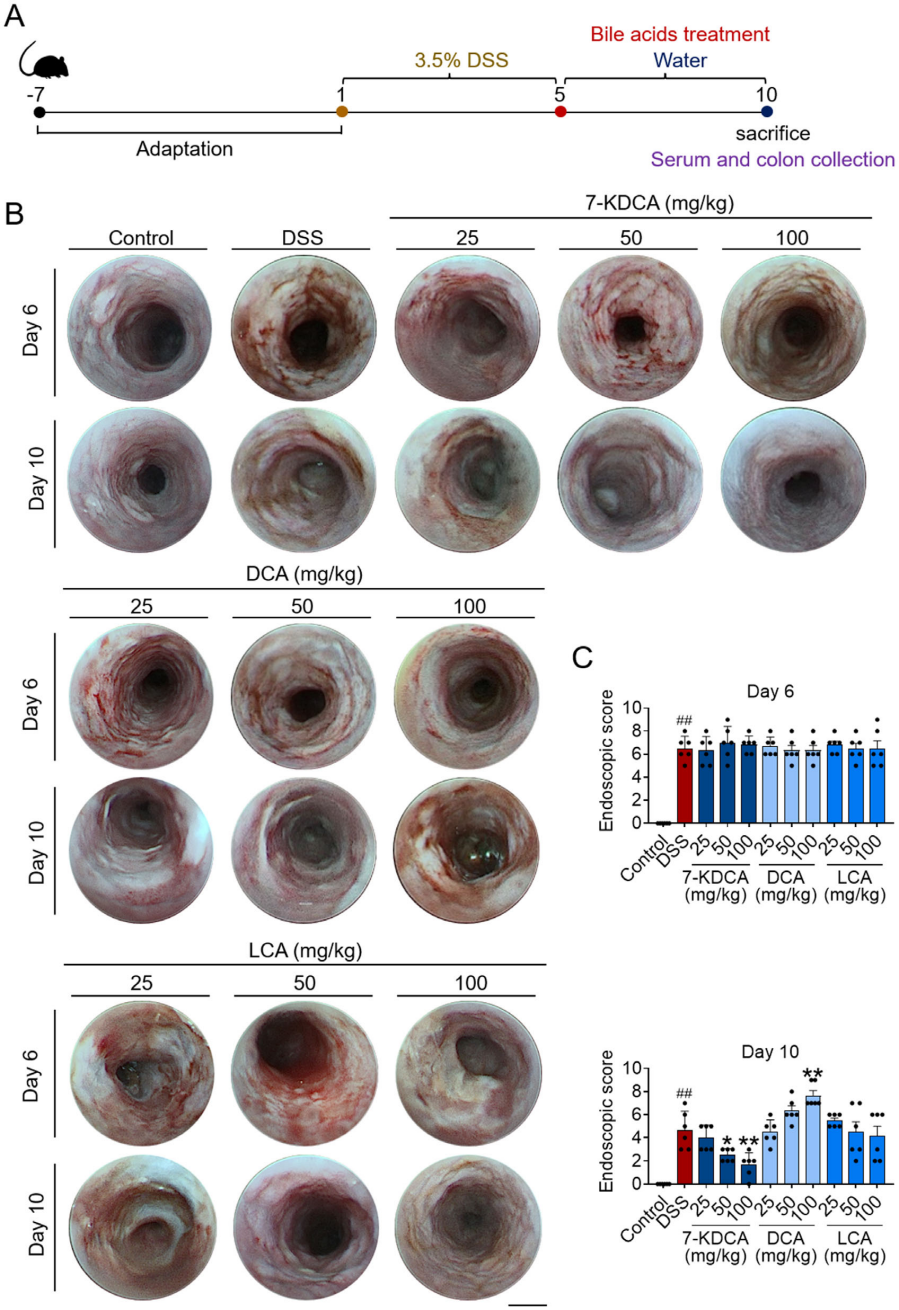
7‐KDCA administration during the recuperative phase from DSS expedites mucosal healing. C57BL/6 mice underwent a 5‐day exposure to 3.5% DSS, succeeded by a subsequent 5 days of water together with vehicle or bile acids. A) Strategy for bile acids treatment during the recovery period of DSS colitis model. B) Representative images displaying regions characterized by overt bleeding and edema. C) Endoscopic score. Data represent mean ± SD (n = 6). ^##^ *p* < 0.01 vs. Control group. ^*^ *p* < 0.05, ^**^ *p* < 0.01 vs. DSS group. 7‐KDCA, 7‐ketodeoxycholic acid; DSS, dextran sulfate sodium; DCA, deoxycholic acid; LCA, lithocholic acid.

To compare the anti‐colitis effects of the exogenous supplementation of 7‐KDCA, DCA, and LCA, we employed a specific model that simulated injury and subsequent restoration of the colonic mucosa, respectively, via the addition of DSS in drinking water and then a return to water alone.^[^ ^16^ ^]^ Colitic mice were orally gavaged with 7‐KDCA, DCA, or LCA (**Figure** **3**A). Notably, replenishment with 7‐KDCA substantially increased the survival rate, inhibited weight loss, lowered the DAI score, alleviated the reduction in colon length, mitigated the intestinal permeability, and decreased the MPO activity and expression of proinflammatory cytokines in the colon of DSS‐challenged mice (Figures S5 and S6A, Supporting Information). It is worth noting that this protective effect of 7‐KDCA was dose‐dependent (Figures S5 and S6A, Supporting Information). Consistent with previous reports, DCA exacerbated colitis while LCA mitigated it (Figures S5 and S6A, Supporting Information).^[^ ^17^, ^18^, ^19^, ^20^, ^21^, ^22^ ^]^

In our endeavor to examine the influence of 7‐KDCA on the recovery of intestinal mucosa, we conducted additional investigations. 7‐KDCA robustly enhanced the expression of proteins related to intestinal mucosal barrier (ZO‐1 and claudin‐1) and factors linked to mucosal restoration (*Occludin*, *Tff3* and *Tgf‐β*) in colitic mice (Figure S6B,C, Supporting Information). Indeed, these mice exhibited enduring bleeding and edema, accompanied by copious and sizable mucosal ulcerations, while mice treated with 7‐KDCA exhibited ameliorated overall endoscopic scores and excellent mucosal healing (Figure 3B,C). Histological analyses of colonic tissues further confirmed the above observation; namely, that mice treated with 7‐KDCA showed less mucosal ulceration and erosion (Figure S7A,B, Supporting Information). Moreover, DCA decelerated while LCA accelerated colonic mucosal healing (Figure 3B,C; Figure S7A,B, Supporting Information). Notably, 7‐KDCA more strongly promoted intestinal healing in colitic mice compared with LCA (Figure 3B,C; Figure S7A,B, Supporting Information). Collectively, these findings imply that 7‐KDCA dramatically accelerates epithelial restitution and repair, with concomitant improvement of intestinal inflammation.

Bile acids have the potential to influence the development and function of host immune cells, including T helper cells expressing interleukin‐17A (TH17 cells), Treg cells expressing interleukin‐10, TH1 cells expressing INF‐γ, and TH2 cells expressing interleukin‐4.^[^ ^23^, ^24^ ^]^ Therefore, we investigated whether 7‐ketodeoxycholic acid modulated the proportions of the Th17, Treg, Th1, and Th2 cells. Results showed that 7‐KDCA (50 mg kg^−1^) had a slight effect on the expression of the Th17‐related cytokine IL‐17 and transcription factor RORγt, Treg‐related cytokine IL‐10 and transcription factor Foxp3, Th1‐related cytokine INF‐γ and transcription factor T‐bet, as well as Th2‐related cytokine IL‐4 and transcription factor GATA3 in the colon tissue of colitic mice, but no significant differences were observed (Figure S8A,B, Supporting Information). Taken together, 7‐KDCA alleviates colitis primarily by accelerating intestinal healing rather than through immune modulation.

To further determine the importance of 7‐KDCA over other bile acids in DSS‐induced colitis, mice were challenged with DSS for 5 days and then treated with 7‐KDCA alone or in combination with LCA or DCA from day 6 until the end of the experiment (Figure S9A, Supporting Information). Similar to 7‐KDCA treatment alone, the combination of 7‐KDCA with LCA or DCA substantially increased the survival rate, increased body weight, lowered the DAI score, alleviated the reduction in colon length, mitigated the intestinal permeability, and decreased the MPO activity in the colon of DSS‐challenged mice (Figure S10, Supporting Information). Moreover, the combination of 7‐KDCA with LCA or DCA robustly accelerated the mucosal repair process in colitic mice, and this finding was supported by endoscopic dynamic monitoring and pathological analyses (Figures S9B,C–S11, Supporting Information). Notably, the combination of 7‐KDCA with LCA did not exhibit a synergistic enhancement effect, and the combination of 7‐KDCA with DCA did not weaken the effect of 7‐KDCA (Figures S9B,C–S11, Supporting Information), suggesting that the function of 7‐KDCA remains unaffected in the presence of other bile acids.

To verify the effect of 7‐KDCA on intestinal wound healing, we employed an alternative method for simulating colonic mucosal injury and repair, incorporating miniaturized endoscopic biopsy‐based mechanical injury and photographic analysis of recovery in the colonic mucosa of mice (Figure S12A,B, Supporting Information).^[^ ^25^, ^26^, ^27^ ^]^ Relative to vehicle control, 7‐KDCA resulted in a notable augmentation of the healing process in colonic mucosal wounds at 72 h (or day 3) after biopsy, while treatment with DCA and LCA had no significant effect (27.85 ± 6.63%, Vehicle; 58.28 ± 11.31%, 7‐KDCA; 27.18 ± 10.52%, DCA; 29.87 ± 5.27%, LCA; Figure S12C, Supporting Information). The above results further confirm that 7‐KDCA may substantially facilitate the process of colonic mucosal repair.

### 2.3 7‐KDCA Drives Colonic Epithelial Cell Migration and Wound Healing Through TGR5

**Figure 4:**
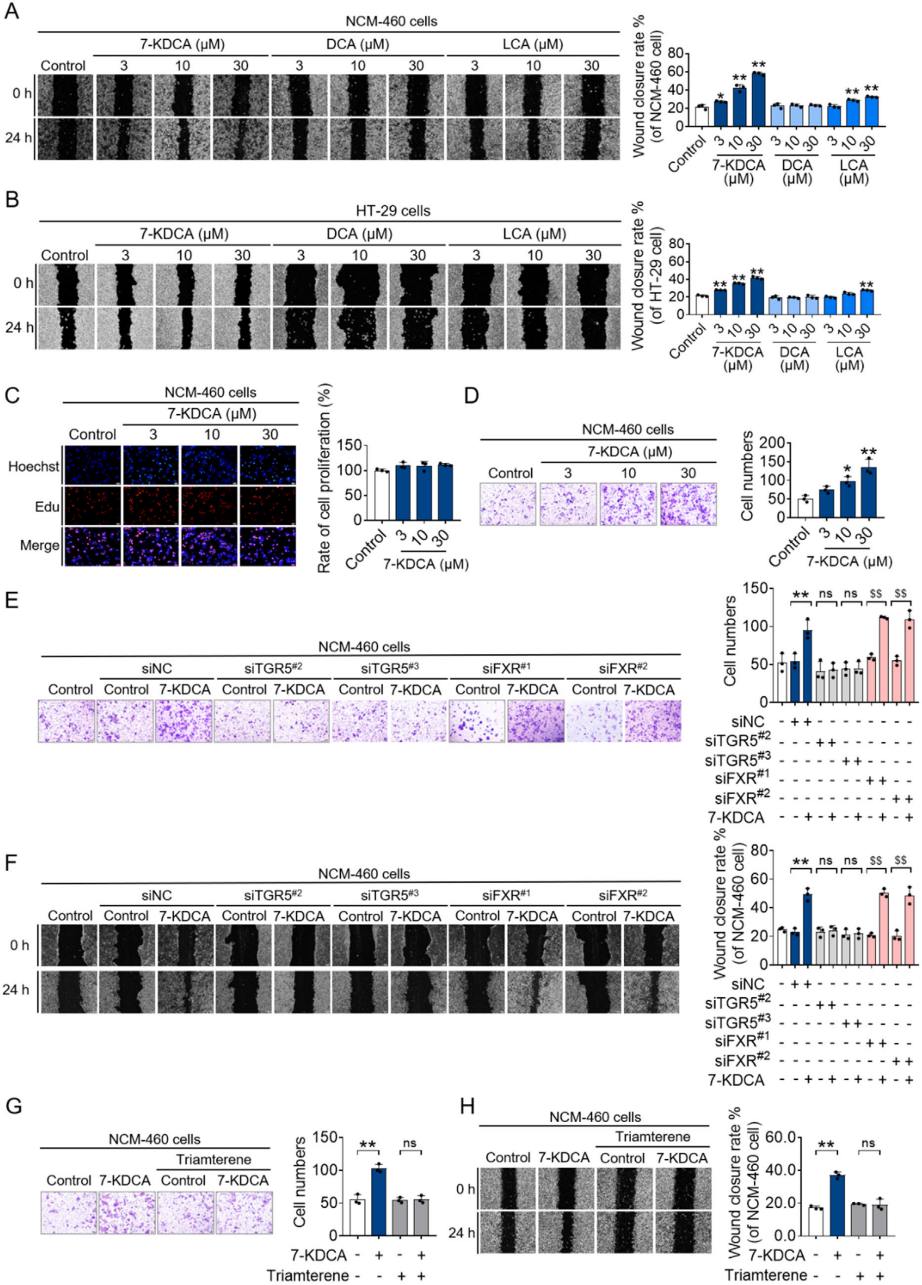
7‐KDCA facilitates migration and healing of human colonic epithelial cells through TGR5. A) Representative images (on the left) and quantitative analysis (on the right) of NCM‐460 cells wound closure, both at baseline and subsequent to 24 h of stimulation with 7‐KDCA, DCA, or LCA. B) Representative images (on the left) and quantitative analysis (on the right) of HT‐29 cells wound closure, both at baseline and subsequent to 24 h of stimulation with 7‐KDCA, DCA, or LCA. C) Percentage of cell proliferation observed at 24 h post 7‐KDCA treatment. D) Rates of cell migration after exposure to 7‐KDCA. E,F) NCM‐460 cells were transfected with siNC or siTGR5 or siFXR, treated with/without 7‐KDCA (10 µm) for 24 h, and subjected to in vitro functional assays, encompassing assessments such as migration (visualized through crystal violet staining) and wound closure (illustrated through a representative scratch assay). G,H) NCM‐460 cells were treated with/without 7‐KDCA (10 µm) in the presence or absence of triamterene (10 µm) for 24 h, and subjected to in vitro functional assays, encompassing assessments such as migration (visualized through crystal violet staining) and wound closure (illustrated through a representative scratch assay). Data represent mean ± SD (n = 3). ^*^ *p* < 0.05, ^**^ *p* < 0.01 vs. Control group or siNC group. ^$$^ *p* < 0.01 vs. siFXR group. Ns, no significant difference. 7‐KDCA, 7‐ketodeoxycholic acid; DCA, deoxycholic acid; LCA, lithocholic acid; siNC, negative control siRNA; TGR5, Takeda G protein‐coupled receptor 5; siTGR5, small interfering RNA of TGR5; FXR, farnesoid X receptor; siFXR, small interfering RNA of FXR.

The self‐healing phenomenon in injured IECs, being crucial for the reconstruction of the intestinal barrier and the regulation of tissue homeostasis, prompted us to explore the effects of the three bile acids on wound repair. DCA or LCA at a concentration of 300 µm induced IEC cytotoxicity in both NCM‐460 and HT‐29 cells as compared to untreated controls (Figure S13, Supporting Information). Remarkably, up to 300 µm of 7‐KDCA did not affect the viability of IECs (Figure S13, Supporting Information), suggesting that this metabolite does not exhibit toxicity in human IECs at physiologically relevant concentrations. Through in vitro scratch wound assay, we found that colon epithelial cells treated with 7‐KDCA had superior healing ability (**Figure** **4**A,B). Importantly, the ability to promote colonic mucosal wound healing was highest in the 7‐KDCA group, followed by the LCA group, and was lowest in the DCA and control groups (Figure 4A,B). Because mucosal wound healing relies upon the synchronized migration and proliferation of IECs,^[^ ^3^ ^]^ our subsequent inquiry delved into the potential impact of 7‐KDCA on cell migration and proliferation in vitro. EdU staining assay indicated that 7‐KDCA treatment led to a slight increase in cell proliferation, yet this was not statistically significant (Figure 4C). 7‐KDCA treatment did, however, significantly enhance the cell migration ability in a concentration‐dependent fashion (Figure 4D), which suggests that the effect of 7‐KDCA in promoting mucosal healing is largely due to its influence on IEC migration.

Farnesoid X receptor (FXR) and TGR5 stand out as the most renowned bile acid‐activated receptors, exerting influence over numerous physiological and pathological processes,^[^ ^28^, ^29^, ^30^ ^]^ thus, we further explored the involvement of these two receptors in the 7‐KDCA‐mediated increase of cell migration and healing capacity. We detected the expression levels of TGR5 in NCM‐460, HT‐29 cells, and primary colonic epithelial cells, and found that TGR5 is highly expressed in all of these colonic epithelial cells (Figure S14, Supporting Information). Furthermore, FXR silencing did not exert a substantial influence on the capacity of 7‐KDCA to enhance cell migration or healing (Figure 4E,F; Figures S15 and S16, Supporting Information). The marked increase in migration and healing promoted by 7‐KDCA was abrogated through TGR5 silencing (Figure 4E,F; Figures S15 and S16, Supporting Information). Similarly, the TGR5 antagonist triamterene completely abolished the enhanced cell migration and healing ability of 7‐KDCA (Figure 4G,H). These results indicate that induction of IEC migration and mucosal wound repair by 7‐KDCA involves TGR5.

**Figure 5:**
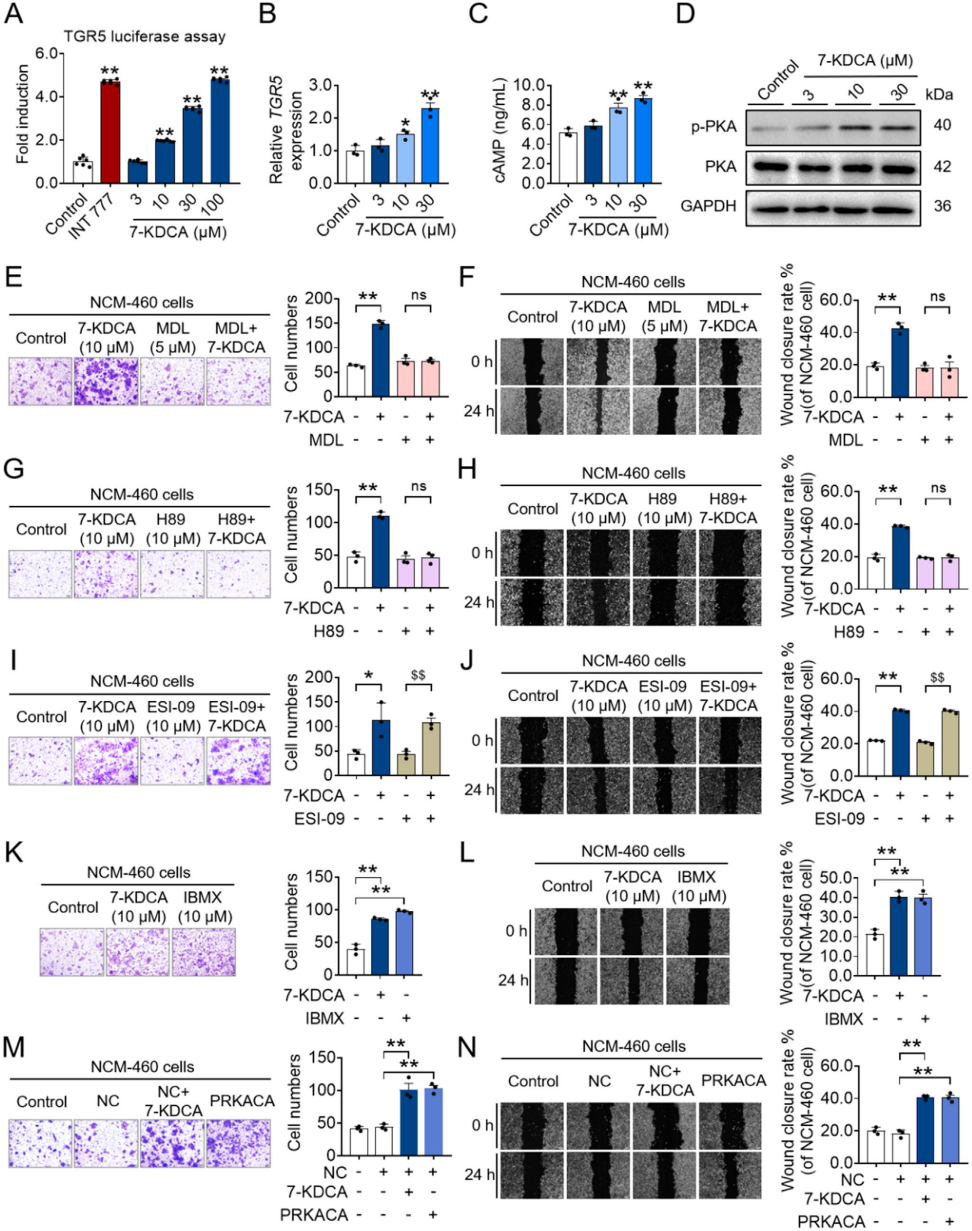
7‐KDCA accelerates migration and healing of human colonic epithelial cells through activation of TGR5. A) TGR5 luciferase reporter activities of 7‐KDCA. B) qPCR analysis of *TGR5* expression in NCM‐460 cells treated with 7‐KDCA. C) cAMP level in NCM‐460 cells treated with 7‐KDCA using ELISA method. D) Western blot analysis of PKA and p‐PKA protein levels in NCM‐460 cells treated with 7‐KDCA. NCM‐460 cells were treated with/without 10 µm 7‐KDCA in the presence or absence of 5 µm MDL12330A E,F), 10 µm H89 G,H), 10 µm ESI‐09 I,J), 10 µm IBMX K,L), or with PRKACA overexpression M,N) for 24 h, and subjected to in vitro functional assays, encompassing assessments such as migration (visualized through crystal violet staining) and wound closure (illustrated through a representative scratch assay). Data represent mean ± SD (n = 3). ^*^ *p* < 0.05, ^**^ *p* < 0.01 vs. Control group or NC group. ^$$^ *p* < 0.01 vs. ESI‐09 group. ns, no significant difference. 7‐KDCA, 7‐ketodeoxycholic acid; TGR5, Takeda G protein‐coupled receptor 5; qPCR, quantitative real‐time polymerase chain reaction; cAMP, cyclic adenosine monophosphate; ELISA, enzyme‐linked immunosorbent assay; PKA, protein kinase A; p‐PKA, phospho‐PKA; MDL, MDL12330A; IBMX, isobutylmethylxanthine; NC, normal control.

We next investigated the activation of human TGR5 by 7‐KDCA or INT‐777, an effective TGR5 agonist, employing a transient transfection assay in HEK293T cells. 7‐KDCA induced activation of human TGR5 in a concentration‐dependent manner (**Figure** **5**A). Some small‐molecule agonists of TGR5 have the capability to elevate the expression of *TGR5*.^[^ ^31^ ^]^ To ascertain whether 7‐KDCA induces *TGR5* expression in addition to activating this receptor, we exposed NCM‐460 cells to 7‐KDCA and measured *TGR5* expression and found that *TGR5* expression was elevated (Figure 5B). In contrast, 7‐KDCA did not affect the expression of *FXR*, *FGF19*, *SHP*, *PXR*, *CAR*, or *VDR* in NCM‐460 cells at tested concentrations (Figure S17, Supporting Information). Our findings confirm that 7‐KDCA, a naturally existing bile acid metabolite, serves as a formidable TGR5 agonist capable of triggering this receptor, thereby precipitating the migration of IECs and facilitating wound healing.

The binding of TGR5 with its corresponding ligands initiates the activation of adenylate cyclase, culminating in a rise in intracellular cyclic adenosine monophosphate (cAMP) levels and subsequent activation of protein kinase A (PKA).^[^ ^32^, ^33^ ^]^ We found that administration of 7‐KDCA notably elevated the intracellular cAMP level and activated PKA in NCM‐460 cells (Figure 5C,D). Importantly, the cell migration and healing induced by 7‐KDCA were entirely nullified upon the addition of MDL12330A, an inhibitor of adenylate cyclase (Figure 5E,F). Within the majority of cells, the modulation of intracellular cAMP signaling is facilitated by two effectors that exhibit affinity for cAMP: PKA and cAMP‐regulated guanine nucleotide exchange factors (which include Epacs1 and 2).^[^ ^34^ ^]^ To ascertain the effector group responsible for eliciting cell migration in response to 7‐KDCA, we subjected NCM‐460 cells to inhibitors specific for either PKAc or Epac1, the latter being more ubiquitously expressed than Epac2, in combination with 7‐KDCA.^[^ ^34^ ^]^ The 7‐KDCA‐induced IEC migration and healing were entirely abolished with the introduction of H89 (a selective PKA inhibitor) but not with ESI‐09 (a selective Epac1 inhibitor) (Figure 5G–J), suggesting that PKA is essential for 7‐KDCA to promote IEC migration and wound healing. Conversely, a reduction in cAMP hydrolysis, facilitated by the phosphodiesterase inhibitor, isobutylmethylxanthine (IBMX), or the overexpression of PRKACA, proved capable of enhancing the migration and reparative processes of IECs (Figure 5K–N; Figure S18, Supporting Information). These results indicate that increased migration and wound healing of colonic epithelial cells by 7‐KDCA is dependent on TGR5 activation.

### 2.4 Calcium Released from the Endoplasmic Reticula Mediates the 7‐KDCA‐Induced Migration and Wound Healing of Colon Epithelial Cells

**Figure 6:**
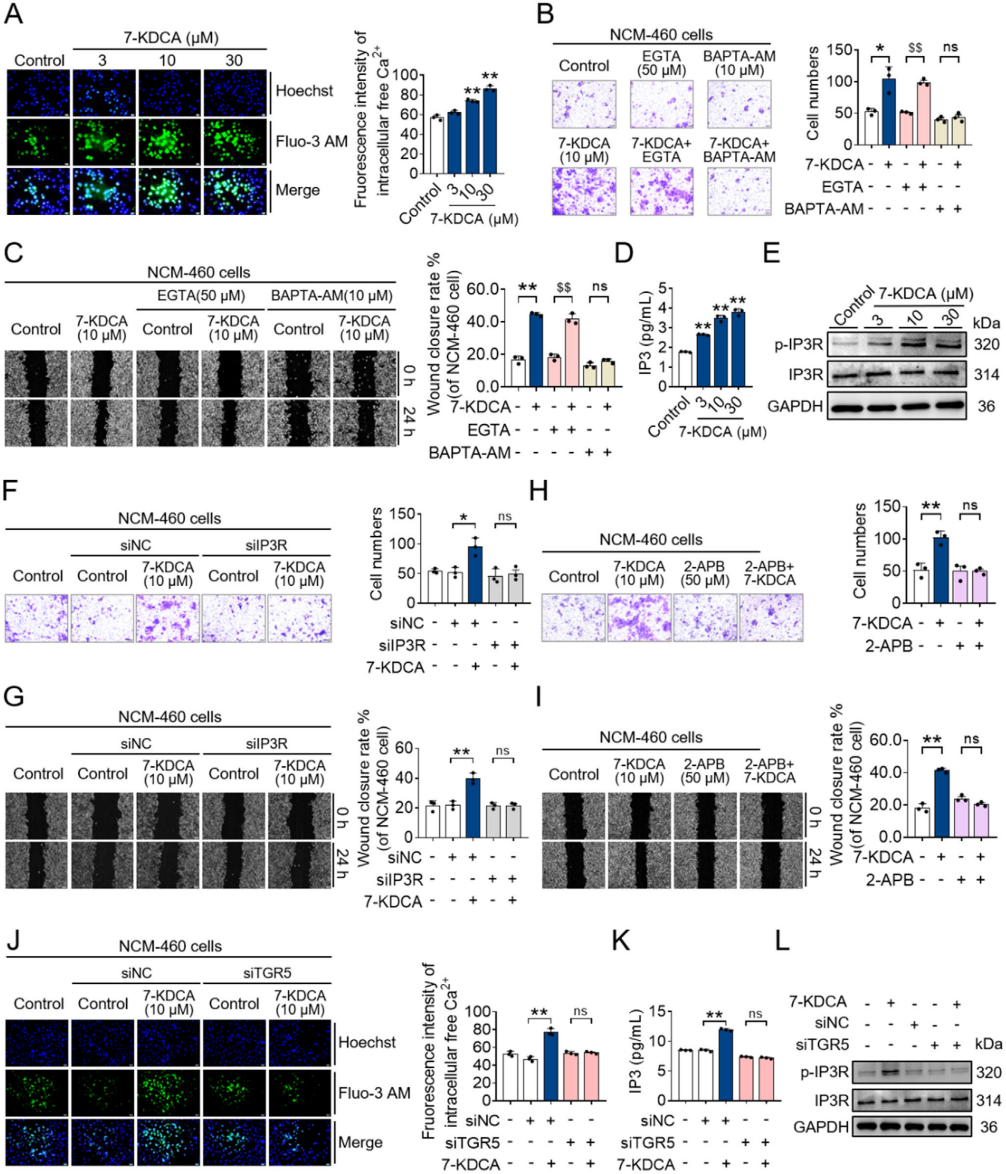
The promotion of migration and healing in colon epithelial cells by 7‐KDCA is mediated through the release of calcium from endoplasmic reticulum. A) The cytoplasmic Ca^2+^ levels in NCM‐460 cells, subjected to treatment with/without 7‐KDCA, were visualized using a fluorescence microscope. B,C) NCM‐460 cells were treated with/without 7‐KDCA in the presence or absence of 50 µm EGTA or 10 µm BAPTA‐AM for 24 h, and subjected to in vitro functional assays, encompassing assessments such as migration (visualized through crystal violet staining) and wound closure (illustrated through a representative scratch assay). D) ELISA analysis of IP3 level in NCM‐460 cells treated with/without 7‐KDCA. E) Western blot analysis of IP3R and p‐IP3R in NCM‐460 cells stimulated with/without 7‐KDCA. F,G) NCM‐460 cells were transfected with siNC or siIP3R, treated with/without 7‐KDCA (10 µm) for 24 h, and subjected to in vitro functional assays, encompassing assessments such as migration (visualized through crystal violet staining) and wound closure (illustrated through a representative scratch assay). H,I) NCM‐460 cells were treated with/without 7‐KDCA (10 µm) in the presence or absence of 2‐APB (50 µm) for 24 h, and subjected to in vitro functional assays, encompassing assessments such as migration (visualized through crystal violet staining) and wound closure (illustrated through a representative scratch assay). J) Ca^2+^ level in the cytoplasm of NCM‐460 cells treated with/without 7‐KDCA after being transfected with siNC or siTGR5 intervention was visualized by a fluorescence microscope. K) ELISA analysis of IP3 level in NCM‐460 cells treated with/without 7‐KDCA after being transfected with siNC or siTGR5 intervention. L) Western blot analysis of IP3R and p‐IP3R protein levels in NCM‐460 cells stimulated with/without 7‐KDCA after transfected with siNC or siTGR5 intervention. Data represent mean ± SD (n = 3). ^*^ *p* < 0.05, ^**^ *p* < 0.01 vs. Control group. ^$$^ *p* < 0.01 vs. EGTA group. Ns, no significant difference. 7‐KDCA, 7‐ketodeoxycholic acid; ELISA, enzyme‐linked immunosorbent assay; IP3, inositol 1,4,5‐trisphosphate; IP3R, IP3 receptor; p‐IP3R, phospho‐IP3R; siNC, negative control siRNA; siIP3R, small interfering RNA of IP3R; TGR5, Takeda G protein‐coupled receptor 5; siTGR5, small interfering RNA of TGR5.

Multiple studies have reported that TGR5 agonism elevates intracellular calcium levels in human colorectal cells and that calcium signaling is a key regulator for cell migration and wound healing, leading us to speculate that intracellular calcium signaling may mediate the aforementioned effects of 7‐KDCA.^[^ ^35^, ^36^, ^37^, ^38^ ^]^ A concentration‐dependent elevation in calcium levels was discerned in NCM‐460 cells upon exposure to 7‐KDCA (**Figure** **6**A). Of note, depletion of extracellular Ca^2+^ with EGTA did not affect the 7‐KDCA‐induced healing and migration in NCM‐460 cells, whereas acute depletion of intracellular Ca^2+^ by BAPTA‐AM, a cell‐permeant Ca^2+^ chelator, completely abolished 7‐KDCA‐induced acceleration of cell migration and wound healing (Figure 6B,C). The endoplasmic reticulum serves as a major intracellular Ca^2+^ store in IECs. Inositol 1,4,5‐triphosphate receptors (IP3Rs), encoded by three genes (*Itpr1*, *Itpr2*, and *Itpr3*), are one of the major Ca^2+^ release channels on the endoplasmic reticulum. IP3 activates IP3Rs to release calcium from the endoplasmic reticulum. In our study, we discovered that 7‐KDCA increased intracellular IP3 levels and activation of IP3Rs in a concentration‐dependent manner (Figure 6D,E). Importantly, small interfering RNA (siRNA)‐mediated knockdown of IP3R or IP3R antagonist 2‐APB completely abolished the 7‐KDCA‐mediated increase in IEC migration and wound healing ability (Figure 6F–I). Xestospongin C is an inhibitor of both IP3R and sarcoplasmic/endoplasmic reticulum Ca2+ ATPase (SERCA) pump of internal stores. Results showed that xestospongin C nearly completely eliminated the increase in cytoplasmic calcium concentration and the promotion of healing ability by 7‐KDCA (Figure S19, Supporting Information). Strikingly, siRNA knockdown of TGR5 nullified the 7‐KDCA‐mediated elevation of intracellular calcium levels and IP3 levels as well as IP3R phosphorylation (Figure 6J–L). These observations indicate that 7‐KDCA facilitates the restoration of mucosal wounds by instigating IEC migration through the induction of TGR5‐mediated calcium release from the endoplasmic reticulum.

### 2.5 The Repair Effect of 7‐KDCA on Intestinal Injury in Mice is Dependent on TGR5‐mediated Calcium Release from the Endoplasmic Reticulum

16S rRNA sequencing data showed that 7‐KDCA obviously altered the structure of gut microbiota, while 7‐KDCA still promoted colonic mucosal healing and alleviated DSS‐induced colitis in microbiota‐depleted mice (Figures S20 and S21, Supporting Information), suggesting that the function of 7‐KDCA in promoting intestinal mucosal healing is independent of the gut microbiota.

Consistent with our in vitro observations, 7‐KDCA enhanced the expression of *Tgr5*, but not *Fxr* and its downstream target genes (*Shp* and *Fgf15*) in the colon tissue of colitic mice (Figure S22A–D, Supporting Information). Colitis status does not alter the expression of *Tgr5* in the colon of mice (Figure S22A, Supporting Information). However, 7‐KDCA enhanced the expression of *Tgr5* in the colon tissue of colitic mice (Figure S22A, Supporting Information), indicating that the expression of *Tgr5* is induced by 7‐KDCA rather than wound healing itself. Moreover, 7‐KDCA increased cAMP levels and activated PKA in the colon of colitic mice (Figure S22E,F, Supporting Information), indicating that 7‐KDCA activated the TGR5 pathway in these mice.

**Figure 7:**
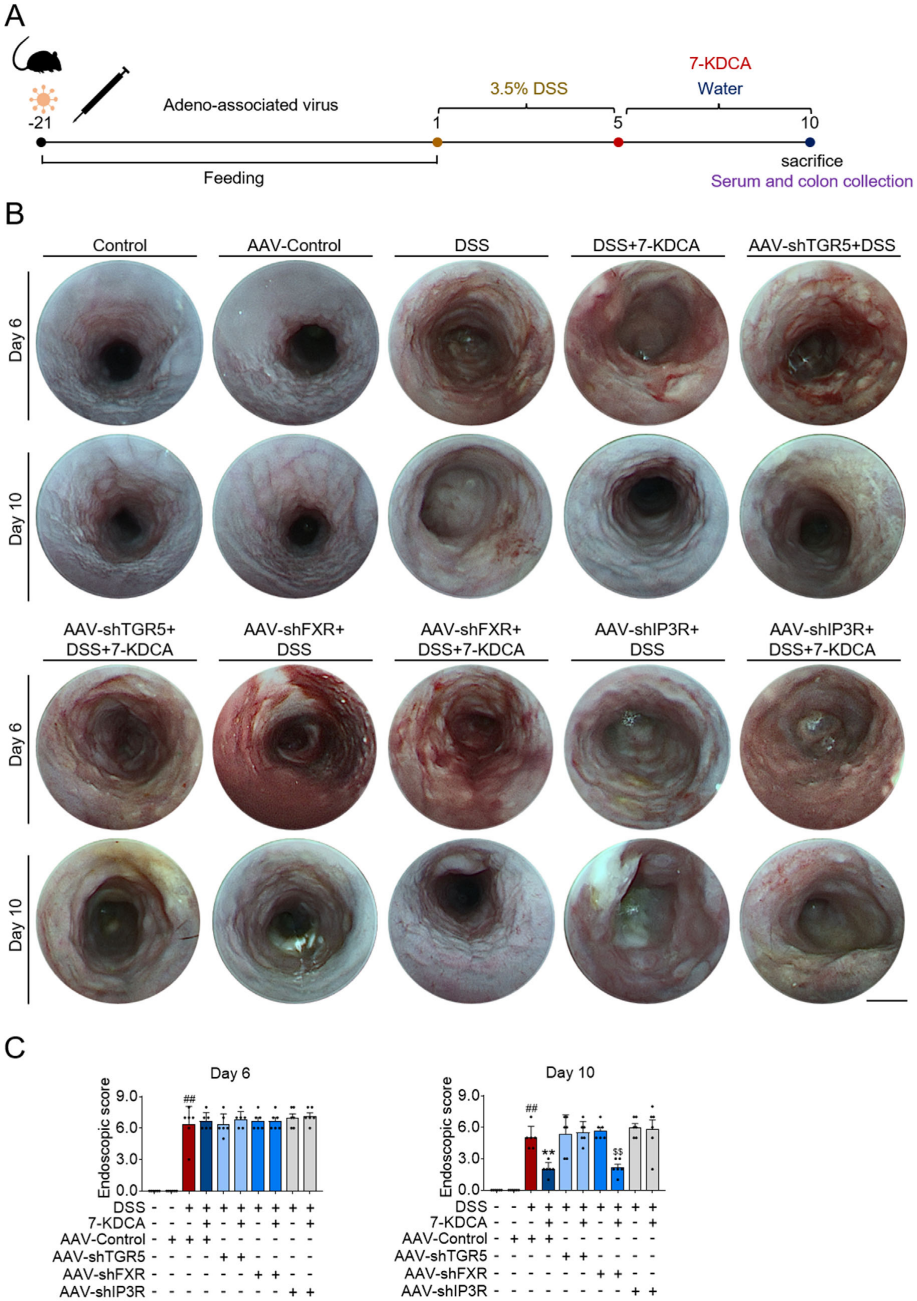
7‐KDCA facilitates the restoration of impaired epithelial mucosa by targeting colonic TGR5‐IP3R signaling. A) On week 3 after enema with lentiviral shRNA targeting TGR5/FXR/IP3R, C57BL/6 mice were exposed to 3.5% DSS (w/v) over a period of 5 days, succeeded by 5 days of water exposure, and intragastrically given bile acids suspension or vehicle control. B) Representative images displaying regions characterized by overt bleeding and edema. C) Endoscopic score. Data represent mean ± SD (n = 6). ^##^ *p* < 0.01 vs. AAV‐Control group. ^**^ *p* < 0.01 vs. DSS group. ^$$^ *p* < 0.01 vs. AAV‐shFXR group. 7‐KDCA, 7‐ketodeoxycholic acid; TGR5, Takeda G protein‐coupled receptor 5; FXR, farnesoid X receptor; IP3R, inositol 1,4,5‐trisphosphate receptor; DSS, dextran sulfate sodium.

To further substantiate the receptor‐dependent role of 7‐KDCA in facilitating intestinal mucosal healing, lentivirus‐mediated short hairpin RNA (shRNA) was used to knockdown the expression of FXR or TGR5 in vivo. The expression of *Fxr* or *Tgr5* in the ileum and colon was dramatically decreased by AAV‐FXR‐shRNA enema or AAV‐TGR5‐shRNA enema, but the expression of *Fxr* and *Tgr5* in the heart, liver, spleen, lungs, kidneys, duodenum, and jejunum was not significantly altered (Figures S23 and S24, Supporting Information). Interestingly, 7‐KDCA was still able to promote intestinal mucosal healing in FXR‐knockdown mice but not in TGR5‐knockdown mice (**Figure** **7**; Figures S25–S27, Supporting Information), further verifying that 7‐KDCA stimulates intestinal mucosal healing via activation of TGR5 in IECs.

Given that 7‐KDCA substantially increased IP3 levels and activated IP3R in the colon of colitic mice (Figure S28, Supporting Information), we next sought to determine whether the disruption of intestinal IP3R interferes with the function of 7‐KDCA in promoting mucosal healing in a murine model of colitis. *Ip3r* expression was markedly attenuated in the ileum and colon of IP3R‐knockdown mice, and the reduction in IP3R began from at least week 3 (Figure S29, Supporting Information). The impact of 7‐KDCA on intestinal mucosal healing was offset in IP3R‐knockdown mice (Figure 7; Figures S25–S27, Supporting Information), underscoring the essential role of IP3R in the effective in vivo promotion of mucosal healing by 7‐KDCA. Critically, 7‐KDCA failed to activate IP3R in TGR5‐knockdown mice (Figure S30, Supporting Information), suggesting that 7‐KDCA accumulation triggers TGR5 activation and subsequent calcium release from the endoplasmic reticulum. These observations collectively support the speculation that 7‐KDCA accelerates intestinal mucosal healing by activating TGR5‐mediated calcium release from the endoplasmic reticulum, thereby alleviating intestinal damage during colitis.

### 2.6 7‐KDCA is Gut‐Restricted and Relatively Safe

Mass spectrometry was used to measure the levels of 7‐KDCA in the intestine to investigate whether 7‐KDCA reaches the intestine after oral administration. Specifically, mice were provided with 3.5% (w/v) DSS solution for a duration of 5 days to initiate colitis. On day 6, they were gavaged with 50 mg kg^−1^ of 7‐KDCA, and colon tissue and serum samples were collected at 0, 0.5, 1, 2, and 4 h post‐administration. Subsequently, mass spectrometry was used to quantify the concentration of 7‐KDCA in the mouse serum, duodenum, jejunum, ileum, and colon. The results showed that the serum concentration of 7‐KDCA peaked at 22.79 ng mL^−1^ at 2 h post‐administration (Figure S31A, Supporting Information). The concentrations of 7‐KDCA in the duodenum and jejunum were relatively high at 0.5 h, reaching 567.49 and 1318.92 µg g^−1^, respectively (Figure S31A, Supporting Information). At 1 h, the concentrations of 7‐KDCA in the ileum peaked at 560.64 µg g^−1^ (Figure S31A, Supporting Information). At 2 h, the concentrations of 7‐KDCA in colon peaked at 123.90 µg g^−1^ (Figure S31A, Supporting Information). Even at 4 h post‐administration, 7‐KDCA levels in the colon remained at 48.86 µg g^−1^ (Figure S31A, Supporting Information). These results indicate that the absorption rate of 7‐KDCA in the blood is quite low, and a large amount of 7‐KDCA is retained in the intestine after oral administration.

After oral administration of 7‐KDCA to mice, although high concentrations of 7‐KDCA were observed in the intestine, no significant upregulation of this metabolite was detected in circulating blood, indicating that 7‐KDCA is not recycled via the enterohepatic circulation nor absorbed into the systemic circulation. In contrast, endogenous TGR5 agonists taurodeoxycholic acid and DCA are detected in the blood of mice.^[^ ^39^ ^]^ After oral gavage of 7‐KDCA, only a small amount of 7‐KDCA was produced in the circulating and portal blood, and the metabolites were mainly retained in the intestine, indicating poor intestinal absorption of 7‐KDCA.

Currently, the therapeutic application of TGR5 agonists is limited due to the side effects associated with their absorption into the systemic circulation.^[^ ^40^, ^41^ ^]^ The TGR5 agonist absorbed throughout the body can reduce bile secretion, promote gallbladder filling, and lead to bile stasis.^[^ ^42^, ^43^ ^]^ In our study, no change in gallbladder weight was observed in mice 4 h after 7‐KDCA gavage compared to the control group (Figure S31B, Supporting Information). Moreover, after 5 consecutive days of 7‐KDCA gavage, no significant changes in gallbladder weight were observed compared to the control group (Figure S31C, Supporting Information). These results indicate that 7‐KDCA does not exhibit one of the primary side effects of non‐intestinal‐restrictive TGR5 agonists, either in acute or chronic conditions.

Without DSS exposure, 7‐KDCA alone had no obvious influence on body weight, colon length, intestinal permeability, and morphological changes in intestinal epithelium (Figure S31D–H, Supporting Information). These findings suggest that 7‐KDCA itself could not significantly disrupt intestinal function.

In summary, 7‐KDCA is a TGR5 agonist that increases *Tgr5* expression and induces colonic mucosal repair without any obvious side effects.

## 3 Discussion

Epithelial barriers at the mucosal and dermal surfaces of the intestine serve as formidable shields, effectively partitioning the internal space from the external environment, thereby safeguarding the underlying tissues from the intrusion of toxins and infectious pathogens. The disruption of epithelial barriers stands as a characteristic phenomenon in a myriad of pathological conditions, encompassing both acute and chronic inflammatory diseases. Following epithelial damage, a process known as restitution, in which normal adjacent IECs migrate in the exposed area to reseal and repair the epithelium, must occur for restoring crucial barrier function.

By conducting a quantitative evaluation of changes in endogenous metabolites, we ultimately ascertained that the levels of 7‐KDCA, DCA, and LCA were decreased in patients with UC. We consistently observed a decrease in 7‐KDCA, DCA, and LCA levels in both the serum and colon of colitic mice. Although secondary bile acids share structural similarities, they demonstrate distinct roles and mechanisms depending on the given context.^[^ ^18^, ^44^, ^45^ ^]^ Research has demonstrated that LCA exerts anti‐colitis effects in various colitis mouse models, while DCA exacerbates the development of colitis.^[^ ^17^, ^18^, ^19^, ^20^, ^21^, ^22^ ^]^ However, few studies have been conducted concerning the impact of 7‐KDCA on UC. Thus, uncertainty persists as to whether 7‐KDCA merely constitutes a metabolic byproduct of DCA or whether 7‐KDCA itself has a crucial physiological and pathophysiological role in maintaining intestinal homeostasis. Our findings demonstrated that 7‐KDCA has an excellent healing effect and is beneficial for the restoration and rebuilding of the colonic epithelium in the course of the mucosal healing process. This observation was corroborated by the empirical data indicating that 7‐KDCA enhanced intestinal mucosal healing in two mouse models of colon injury. It is worth nothing that the wound‐healing effect was greatest in the 7‐KDCA group, followed by LCA group, and was lowest in the DCA and control groups. Considered cumulatively, our findings are the first to identify the role of 7‐KDCA in fostering colonic mucosal wound repair.

Upon further analysis of colon epithelial cells treated with 7‐KDCA, we found a significant increase in migration and healing ability, which impelled us to explore a potential 7‐KDCA‐dependent mechanism(s) for promoting epithelial wound healing. Among all bile acid receptors, we found that only 7‐KDCA increased *TGR5* expression and activated TGR5, indicating that TGR5 may be involved in the promotion of IEC migration and mucosal healing by 7‐KDCA. Given that TGR5 and FXR are the most famous bile acid‐activated receptors, orchestrating a multitude of the gut homeostatic functions of bile acids,^[^ ^28^, ^29^, ^30^ ^]^ we sought to examine the effect of the in vivo and in vitro of TGR5 knockdown and FXR knockdown on the mucosal healing function promoted by 7‐KDCA. The results demonstrated that although FXR was not critical to the accelerated mucosal healing induced by 7‐KDCA, TGR5 was, indicating that TGR5 signaling plays a pivotal role in the epithelial repair promoted by 7‐KDCA.

In vitro studies suggest that TGR5 inhibitors have no significant effect on cell migration ability, but they seem to have some impact in vivo, which presents a contradiction. This discrepancy may be due to the lack of TGR5 agonists in in vitro experiments, rendering the TGR5 inhibitors ineffective. In contrast, in vivo, the presence of endogenous TGR5 agonists allows the TGR5 knockdown to exert some effect.

Although activation of TGR5 has been proposed to promote an increase in intracellular calcium levels,^[^ ^35^ ^]^ the presence of functional crosstalk between bile acids and calcium signaling in the regulation of intestinal mucosal healing has not yet been explored. A plethora of evidence indicates that calcium signaling is essential to cell migration.^[^ ^36^, ^37^, ^38^ ^]^ We observed that 7‐KDCA induced an increase in calcium levels in IECs, which could aid in directing and coordinating IEC migration and wound repair. As expected, the impact of 7‐KDCA on intestinal mucosal healing was counteracted in IP3R‐knockdown mice, indicating that 7‐KDCA operates through releasing calcium from the endoplasmic reticulum to support intestinal cell migration and wound closure.

While our study has elucidated the mucosal healing‐promoting effects of 7‐KDCA and its underlying mechanisms, it is important to consider the microbial origins of this metabolite. Accumulating evidence indicates that gut microbiota can catalyze the production of various novel bile acids, including 7‐KDCA, through hydroxysteroid dehydrogenases.^[^ ^46^, ^47^, ^48^ ^]^ This microbial metabolic potential has been further highlighted by recent discoveries of specific bacterial strains (e.g., *Clostridium bolteae* and *Bifidobacterium animalis* subsp*. lactis*) capable of producing therapeutic metabolites.^[^ ^49^, ^50^ ^]^ Although the current study focused on the functional characterization of 7‐KDCA, the identification of its microbial producers represents an important direction for future research. The lack of corresponding fecal samples precludes comprehensive microbiome characterization in our current study cohort. Future investigations will require acquisition of properly preserved stool specimens for shotgun metagenomic profiling coupled with functional validation through bacterial cultivation and colonization studies to elucidate microbial sources of 7‐KDCA. These studies promise to both delineate the biological origins of this metabolite and provide novel microbiota‐directed therapeutic approaches for UC.

Our data from in vitro and in vivo investigations have revealed a pivotal role of 7‐KDCA in fostering intestinal epithelial healing. Furthermore, we found that 7‐KDCA accumulation triggers TGR5 activation and subsequent calcium release from the endoplasmic reticulum. The unexpected function of 7‐KDCA in coordinating TGR5‐mediated intestinal mucosal healing offers fresh mechanistic insights into the intricate interplay between endogenous metabolite signaling and the functional of IECs and could potentially inform novel therapeutic approaches in intestinal regeneration based on endogenous metabolite mimetics.

## 4 Experimental Section

### Human Subjects

The research received approval from the Ethics Committee of Jiangsu Province Hospital of Chinese Medicine on August 1, 2022 (ethics number: 2022NL‐135‐02). All participants provided written informed consent. Blood was harvested from veins of each participant, and preserved in a centrifuge tube at 37 °C. Following coagulation, the blood specimens from individuals with UC (n = 32) and those from HC (n = 14) underwent centrifugation to acquire serum (online Table S2, Supporting Information for healthy controls and patient characteristics).

### Untargeted Metabolomics Analysis

Sera (100 µL) were precipitated with 500 µL of methanol‐acetonitrile‐water (2:2:1, v/v/v), and centrifuged for 15 min. The supernatants were subjected to evaporation until dryness. Subsequently, the desiccated product was reconstituted in a 150 µL solution of 50% methanol in water and subjected to centrifugation for 15 min. The resultant supernatants were transferred into an injection bottle for subsequent detection. UPLC‐QTOF‐MS/MS analysis was employed using a 1290 Infinity system coupled with a 6545 quadrupole time‐of‐flight system, operating in both in positive and negative modes. The mobile phase consisted of 0.1% formic acid‐water (v/v; A) and 0.1% formic acid‐acetonitrile (v/v; B). A constant flow rate of 0.3 mL min^−1^ was sustained, with the injection volume set at 2 µL. The optimal gradient elution protocol for serum samples unfolded as follows: 0 to 2 min, 5% B; 2 to 5 min, 5 to 40% B; 5 to 10 min, 40 to 60% B; 10 to 15 min, 60 to 55% B; 15 to 23 min, 55 to 95% B; 23 to 30 min, 95 to 5% B. Chromatographic raw data underwent processing through Agilent MassHunter Profiler software, enabling automated identification of peaks corresponding to metabolic components. Subsequently, the processed data was imported into MetaboAnalyst 5.0 (https://www.metaboanalyst.ca/) for multivariate statistical analysis. Annotation of the detected compounds was performed using HMDB (http://www.hmdb.ca), MassBank (http://www.massbank.jp), and LIPID MAPS (https://www.lipidmaps.org/), leveraging primary and secondary mass spectrum information. Ultimately, the creation of heatmaps was executed utilizing the MetaboAnalyst 5.0 platform.

### Bile Acid Measurements

Serum or tissue samples were precipitated with pre‐cooled methanol and isotope‐labeled internal standards. After being centrifuged, the upper supernatants were subjected to evaporation until dryness. The desiccated substance was subsequently reconstituted in 50% methanol aqueous solution and centrifuged. Subsequently, the supernatants were transferred into an injection vial for detection. UPLC separation was performed using a Waters ACQUITY UPLC I‐Class system. The mobile phase comprised solvent A (0.1% aqueous formic acid) and solvent B (methanol). A constant flow rate of 0.3 mL min^−1^ was sustained, with the injection volume set at 2 µL. The column and autosampler temperatures were maintained at 45 and 8 °C, respectively. The chromatographic gradient established for mobile phase B unfolded as follows: 0 to 6 min: 60% to 65% B; 6 to 13 min: 65% to 80% B; 13 to 13.5 min: 80% to 90% B; 13.5 to 15 min: 90% B. Finally, quantitation was attained through MS detection in a negative ion mode employing a 5500 QTRAP mass spectrometer (AB SCIEX). The conditions of 5500 QTRAP ESI source conditions were as follows: the source temperature was set at 550 °C; ion source gas1 and ion source gas2 were both maintained at 55; and the curtain gas stood at 40. The chromatographic peak area and retention time were computed utilizing Multiquant software. Subsequently, a standard curve was formulated and applied to determine the level of bile acids in serum and colon.

### Mice

Male C57BL/6J mice (10–12 weeks old, weighing 25–28 g) were procured from Jiangsu Huachuang Xinnuo Pharmaceutical Technology Co., Ltd (Taizhou, China). Mice were maintained at a temperature of 25 °C under a 12‐h light/dark cycle. All procedures pertaining to animal experiments were meticulously adhered to in accordance with animal care laws and guidelines, and approved by the ethics committee of China Pharmaceutical University (approval number: 2022‐09‐040).

### DSS‐Induced Murine Colitis and Recovery Model

To investigate the function of three bile acids, mice were randomly allocated into eleven groups, including control group, DSS group and nine administration groups. C57BL/6J mice were provided with 3.5% (w/v) DSS solution for a duration of 5 days to initiate colitis, succeeded by a subsequent 5‐day period with regular drinking water for recovery. Mice were administered 7‐KDCA (25, 50, 100 mg kg^−1^) or DCA (25, 50, 100 mg kg^−1^) or LCA (25, 50, 100 mg kg^−1^) or vehicle for 5 days. To evaluate the extent of DSS‐induced intestinal injury and inflammation, the daily evaluation encompassed the ratio of body weight loss, consistency of stool, and occurrence of rectal bleeding was assessed. The DAI score was computed as the average of these three scores, spanning from 0 (indicating no inflammation) to 4 (reflecting severe colitis). At the end of the experiment, mice were euthanized and colon tissue were harvested. Hematoxylin‐Eosin (H&E) staining was conducted on sections of Swiss roll mounts encompassing the entire colon (with a thickness of 6 µm) to quantify colonic mucosal injury. The percentage of injury/ulceration was computed as a ratio of the length of injured/ulcerated area (≥50% crypt loss) in relation to the entire colon length.^[^ ^26^ ^]^

For the safety evaluation study, mice were allocated into two groups: 1) control; and 2) 7‐KDCA (50 mg kg^−1^). Mice were administered with or without 7‐KDCA for 5 days.

### Knockdown Test in Vivo

AAV‐shRNA‐Tgr5/Fxr/Ip3r or AAV‐shRNA‐scrambled control (Synbio Technologies, Suzhou, China) was slowly injected into the mouse colon (≈4 cm from the proximal anus) through a flexible hose. The mice were kept upside down for 3 min to prevent spillover. Finally, all mice were put back into the cage for further feeding to the end of experiment. The knockdown efficiency of Tgr5, Fxr, and Ip3r was assessed in tissues through qPCR, as delineated in the procedures for RNA isolation and qPCR.

### In Vivo Wounding of Colonic Mucosa

A biopsy‐induced injury to the colonic mucosa in mice was executed following previously delineated procedures.^[^ ^25^ ^]^ Briefly, mice were anesthetized utilizing isoflurane. Biopsy‐induced injuries of the colonic mucosa were generated adjacent to the mesenteric artery employing a high‐resolution, miniaturized colonoscope system fitted with a biopsy forceps (Colorview Veterinary Endoscope, Karl Storz). Images capturing the compromised mucosa were acquired at 24 and 72 h post‐injury for the assessment of the percentage of wound closure. Biopsy wound images were analyzed in a blinded manner to minimize observer bias.

### Colonoscopy

The macroscopic advancement of colitis was evaluated through endoscopic examination of the left colon and rectum, according to a standardized protocol. Individual mice were evaluated by an operator blinded to experiments, and endoscopic scores were calculated using an established semi‐quantitative scoring system that takes into account perianal observations, wall transparency, intestinal bleeding, focal lesions (such as erosions and ulcers), intestinal strictures, flat and/or protruding masses, perforations, and animal death occurring during the procedure.^[^ ^51^, ^52^ ^]^

### FITC‐Dextran Intestinal Permeability Assay

Mice were orally administered 44 mg/100 g body weight of FITC‐dextran solution (Sigma‐Aldrich, # 46944‐500MG‐F). Following a 4 h interval post‐gavage, mice were euthanized and serum samples were obtained. The concentration of FITC dextran in the serum was measured at wavelengths of 485/530 nm.^[^ ^53^ ^]^

### RNA Isolation and qPCR

Total RNA was extracted from mouse colon, followed by the synthesis of cDNA utilizing the HiScript reverse transcriptase system. Subsequently, PCR assay was executed using the Hieff qPCR SYBR Green Master Mix. Primer sequences were enumerated in online Table S3 (Supporting Information). The mRNA expression of each gene was standardized based on GAPDH levels.

### Cell Culture

Human normal colon epithelial cell line (NCM‐460) was furnished by EK‐Bioscience Biotechnology Co., Ltd (China), and human adenocarcinoma colorectal cell line (HT‐29) was procured from Chinese Academy of Sciences (China). They were cultured in RPMI 1640 medium supplemented with 10% FBS. Before the experiment, 7‐KDCA, LCA, and DCA were diluted in DMSO.

### Cell Viability Assay

MTT reduction method was employed to assess the viability of NCM‐460 and HT‐29 cells exposure to 7‐KDCA or DCA or LCA. Cells were seeded into 96‐well plates and subjected to incubation with varying concentrations of 7‐KDCA or DCA or LCA. Then, MTT solution was introduced into each well, followed by an incubation period of 4 h. The supernatant was meticulously removed, and the sediment was subsequently dissolved in DMSO. The optical absorbance at 570 nm was measured.

### Wound Healing Assay

The bottom of a 12‐well plate was annotated to ascertain the position of the scratch wound. Subsequently, cells were seeded into 12‐well plates. Upon the establishment of the cellular monolayer, the apex of a 200‐µL pipette was employed to uniformly scrape the cell monolayer, thereby generating the wound space. Following the rinsing of the monolayer with PBS, the image was captured and documented as the 0 h reference point. Then, fresh complete medium was added to the well. After 24 h, the closure of the wound was documented.

### Migration Experiment

Cells were seeded into a Transwell chamber consisting of 12 wells. Upon cells firmly adhering to the wall, a serum‐free medium was introduced into the upper cavity, while a 10% FBS medium, serving as a chemical attractant, was administered to the lower cavity. Following a 24 h incubation period, the cells within the upper cavity were cleared. The cells that persisted at the base of the filter were immobilized using a 4% paraformaldehyde solution and subsequently stained with 0.1% crystal violet. Subsequently, the migrated cells in the lower chamber were observed through a microscope.

### EdU Staining Assay

Cells were seeded into a 12‐well plate. Upon adhering to the surface, cells underwent an incubation period with varying concentrations of 7‐KDCA or DCA, or LCA for a duration of 24 h. Subsequently, the cells underwent incubation with EdU, followed by fixation using 4% formaldehyde solution. Next, cells were subjected to exposure to glycine and Triton X‐100. Subsequently, cells underwent staining with reaction solution and Hoechst 33342 solution. Ultimately, cellular images were captured utilizing a fluorescence microscope.

### TGR5 Luciferase Assay

The HEK293T cell line was cultured in an incubator with 37 °C and 5% CO_2_, utilizing DMEM supplemented with 10% FBS. The pCRE‐luc plasmid (20 ng/hole) was transfected into HEK293T cells using lipofectamine 3000 transfection reagent (Thermo Fisher). Then, cells were subjected to incubation with 0.2% DMSO as vehicle, INT‐777 as positive control, and 7‐KDCA as specified concentration for 24 h. Following a 24 h incubation period, the culture medium was refreshed, concurrently introducing various concentrations of compound solutions. After 24 h, the Luciferase Reporter Assay System (Promega Madison, WI, USA) was employed following established procedures.^[^ ^54^ ^]^

### Quantification of cAMP Level

The concentration of cAMP in intracellular and colon tissue were assayed through ELISA, following the guidelines provided by the manufacturer.

### Western Blot Analysis

Proteins within lysates derived from colon tissue and NCM‐460 cells were subjected to separation via SDS‐PAGE and subsequently transferred onto NC membranes. The membranes underwent blocking with 5% defatted milk in TBST for 2 h. Subsequently, they were incubated with specific primary antibodies overnight at 4 °C, followed by incubation with the suitable secondary antibody for 2 h at room temperature. GAPDH antibody served as the internal reference to validate the uniform loading of proteins. The protein bands were visualized utilizing enhanced chemiluminescent reagent and subsequently analyzed through Image J software.

### Measurement of Calcium Ions

Alterations in intracellular calcium were identified employing the Fluo‐3 AM kit (Beyotime Institute of Biotechnology, Shanghai, China).^[^ ^55^ ^]^ The NCM‐460 cells underwent incubation with a medium containing 1 µm Fluo‐3AM at 37 °C for 30 min in the absence of light, followed by incubation with Hoechst at 37 °C for 20 min. The images were captured using a fluorescence microscope, and the data were subsequently analyzed to portray MFI utilizing Image J software. MFI served as the metric for assessing the magnitude of Ca^2+^ efflux.

### Gene‐Silencing by siRNA

FXR/TGR5/IP3R silencing was accomplished through specific siRNA (Synbio Technologies, Suzhou, China). Before the transfection process, NCM‐460 cells were seeded into 24‐well plates to attain an approximate 70% confluence. Subsequently, NCM‐460 cells underwent transfection with siRNA, employing Lipo6000 (Beyotime Biotechnology, Shanghai, China) in accordance with the guidelines provided by the manufacturer.

### Statistical Analysis

Statistical analysis was executed utilizing GraphPad Prism 8.0. Data were expressed as the mean ± S.D. Student's t‐test and one‐way analysis of variance, followed by a post‐hoc Tukey's test, were employed to discern the statistical variation among two or more groups. A value of *p* < 0.05 was deemed as statistically significant.

## Conflict of Interest

The authors declare no conflict of interest.

## Author Contributions

Y.D., Y.X., F.J., and Z.W. designed and supervised the study. J.Z., Y.G., and Y.Z. Collected and analyzed the data. J.Z., W.X., M.Z., and L.X. conducted animal, cell cultivation, and mass spectrometry experiments. Y.D., Y.X., and J.Z. wrote the manuscript. Y.D., Y.X., and J.Z. contributed to text revision and discussion. Y.D. was responsible for the overall content of this study. All authors discussed the results and approved the manuscript.

## Supporting information



Supporting Information

## Data Availability

The data that support the findings of this study are available from the corresponding author upon reasonable request.
