# Supplementary material for: 7‐Ketodeoxycholic Acid Promotes Colonic Mucosal Healing by Inducing Calcium Release from Endoplasmic Reticulum via the TGR5‐IP3R Pathway
Source: Adv Sci (Weinh). 2025 Sep 15;12(42):e07953. doi: 10.1002/advs.202507953 (PMC12622453; doi:10.1002/advs.202507953)
Supplement: Supplementary file 1 — Supporting Information [file ADVS-12-e07953-s001.docx]

**Supporting Information**

**7-Ketodeoxycholic Acid Promotes Colonic Mucosal Healing by Inducing Calcium Release from Endoplasmic Reticulum *via* the TGR5-IP3R Pathway**

*Jing Zhang^#^, Feng Jiang^#^, Wenxin Xia, Yilei Guo, Yanrong Zhu, Mianjiang Zhao, Lingzi Xiao, Zhifeng Wei, Yufeng Xia,* and Yue Dai**

^#^ These authors equally contributed to this paper.

J. Zhang, Y.-F. Xia

Department of Pharmacognosy

School of Traditional Chinese Pharmacy

China Pharmaceutical University, Nanjing, China

Nanjing 211198, P. R. China

E-mail address: [yfxiacpu@126.com](mailto:yfxiacpu@126.com)

W.-X. Xia, Y.-L. Guo, Y.-R. Zhu, M.-J. Zhao, L.-Z. Xiao, Z.-F. Wei, Y. Dai

Department of Pharmacology of Chinese Materia Medica

School of Traditional Chinese Pharmacy

China Pharmaceutical University, Nanjing, China

Nanjing 211198, P. R. China

E-mail address: [yuedaicpu@cpu.edu.cn](mailto:yuedaicpu@cpu.edu.cn)

F. Jiang

Affiliated Hospital of Nanjing University of Chinese Medicine, Nanjing, China

Nanjing 210029, P. R. China

E-mail address: jfacer68@hotmail.com

**Supplemental Figure Legends**

**
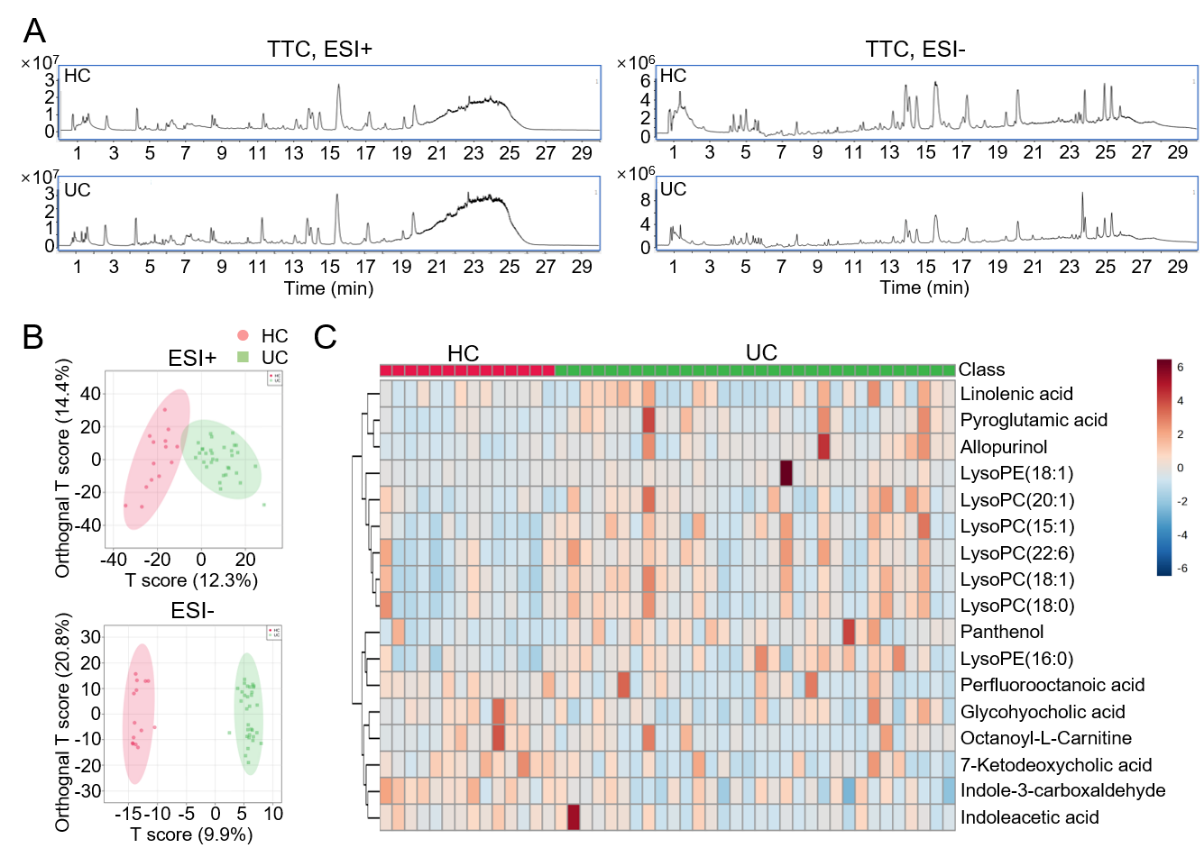
**

**Figure S1.** Metabolic alternations in UC patients. Typical total ion chromatograms (A) and the OPLS-DA score plots (B) of serum obtained from HC (n=14) and UC group (n=32) in positive and negative ion mode. (C) Heatmap was constructed to visually depict the alterations in the concentrations of differential metabolites in serum between HC (n=14) and UC patients (n=32). Each column represents a subject. UC, ulcerative colitis; OPLS-DA, orthogonal partial least squares-discriminant analysis; HC, healthy controls.

**
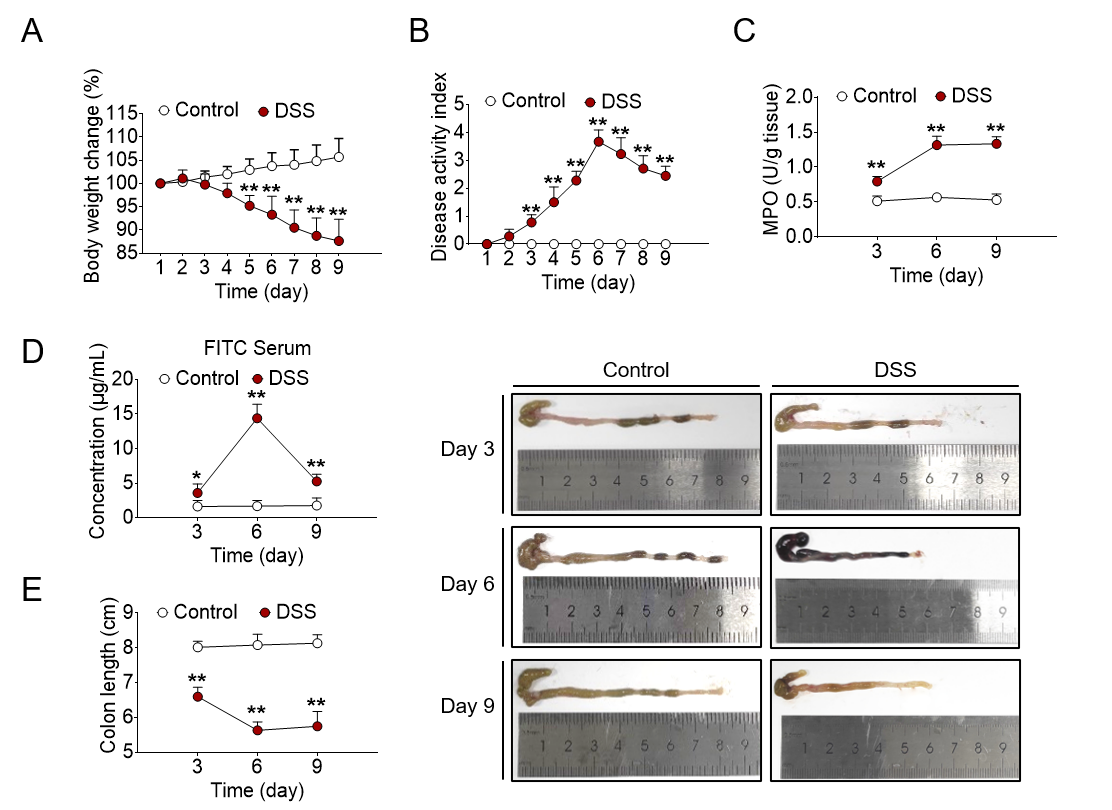
**

**Figure S2.** The severity of DSS-induced mouse colitis at different stages. Mice were treated either with or without 3.5% DSS for 5 days, succeeded by 4 days of water exposure. Colon and serum samples were harvested on days 3, 6 and 9 after DSS consumption. (A) Percentage change in body weight. (B) DAI score. (C) MPO activity in colon tissues. (D) Relative fluorescence intensity of FITC-dextran in serum. (E) Colon length. Data represent mean ± SD (n = 6). *P < 0.05, **P < 0.01 vs. control group. DSS, dextran sulfate sodium; DAI, disease activity index; MPO, myeloperoxidase; FITC, fluorescein isothiocyanate.

**
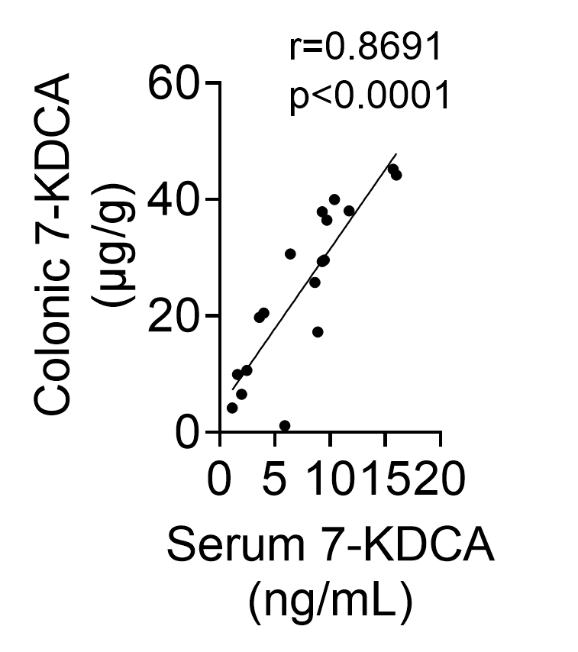
**

**Figure S3.** The level of 7-KDCA in serum is positively correlated with the level of 7-KDCA in the colon. Data represent mean ± SD (n = 18). 7-KDCA, 7-ketodeoxycholic acid.

**
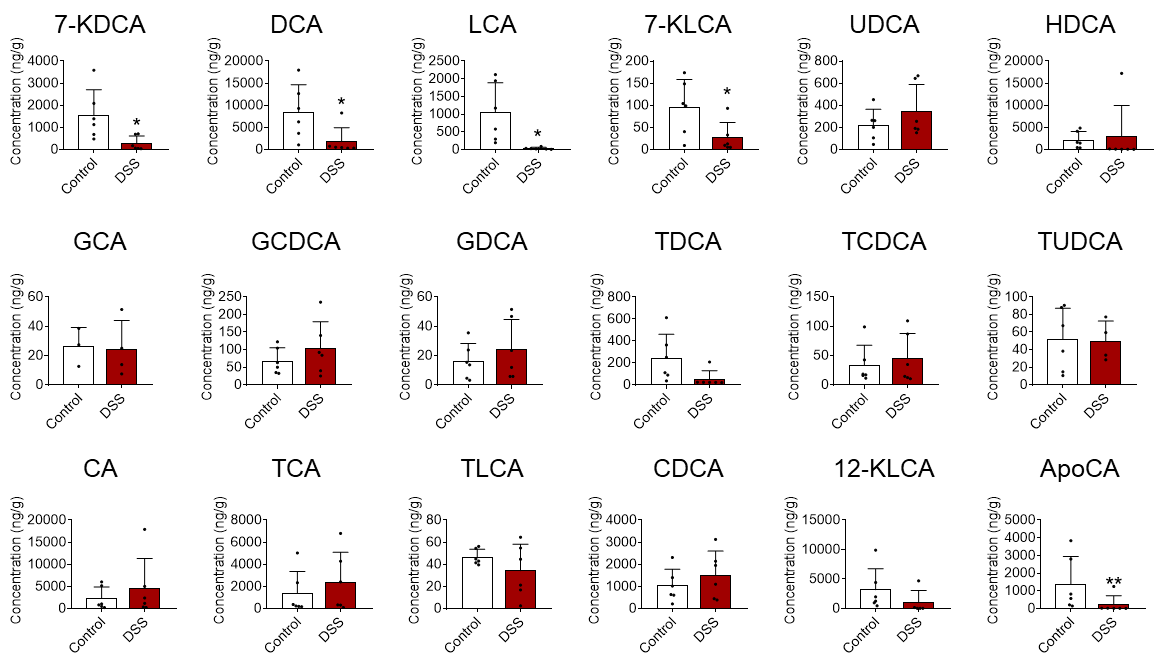
**

**Figure S4.** The levels of 7-KDCA, DCA and LCA are decreased in the colon tissue of mice with experimental colitis. Mice were treated either with or without 3.5% DSS for 5 days, succeeded by 5 days of water exposure. Data represent mean ± SD (n = 6). *P < 0.05 vs. control group. 7-KDCA, 7-ketodeoxycholic acid; DCA, deoxycholic acid; LCA, lithocholic acid; DSS, dextran sulfate sodium.

**
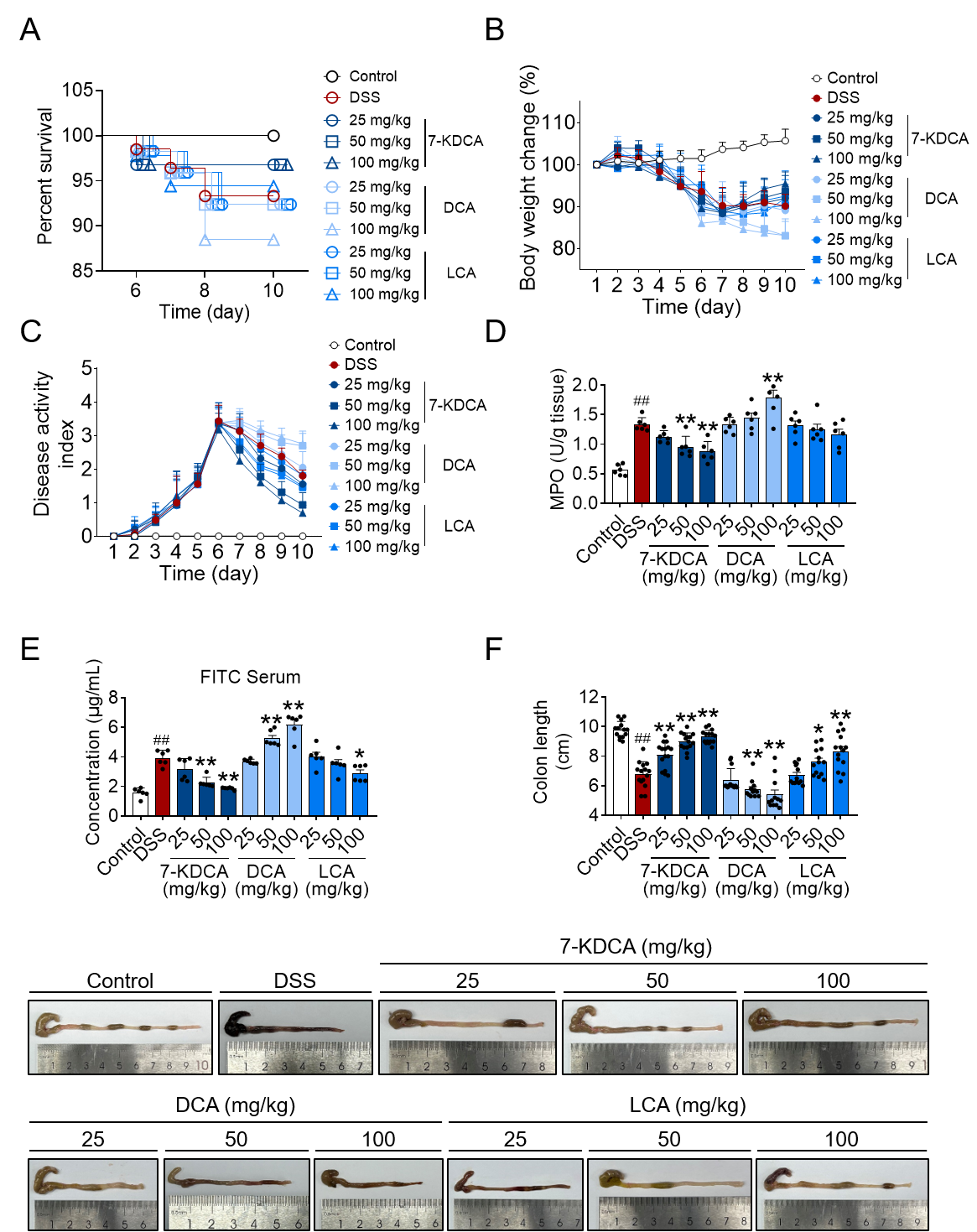
**

**Figure S5.** 7-KDCA administration during the recuperative phase from DSS mitigates the severity of colitis. C57BL/6 mice underwent a 5-day exposure to 3.5% DSS, succeeded by a subsequent 5 days of water together with vehicle or bile acids. Comparison of biochemical parameters of mice among multiple groups. (A) Survival rate (d, days). (B) Percentage change in body weight. (C) DAI score. (D) MPO activity in colon tissues. (E) Relative fluorescence intensity of FITC-dextran in serum. (F) Colon length. Data represent mean ± SD (n = 14-16). ^##^P < 0.01 vs. Control group. *P < 0.05, **P < 0.01 vs. DSS group. 7-KDCA, 7-ketodeoxycholic acid; DSS, dextran sulfate sodium; DCA, deoxycholic acid; LCA, lithocholic acid; DAI, disease activity index; MPO, myeloperoxidase; FITC, fluorescein isothiocyanate.

**
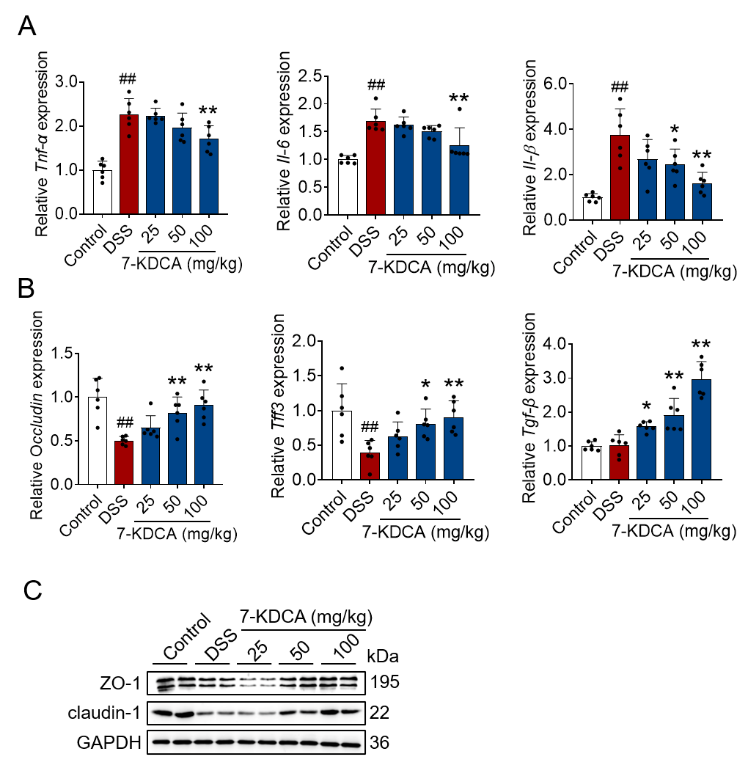
**

**Figure S6.** 7-KDCA accelerates epithelial restitution and repair, with concomitant improvement of intestinal inflammation. (A) The mRNA levels of *Tnf-α*, *Il-6* and *Il-1β* in colon tissues was assessed through qPCR analysis. (B) The mRNA levels of *Occludin*, *Tff3* and *Tgf-β* in colon tissues was assessed through qPCR analysis. (C) Western blot analysis of ZO-1 and claudin-1 protein levels in colon tissues. Data represent mean ± SD (n = 6). ^##^P < 0.01 vs. Control group. *P < 0.05, **P < 0.01 vs. DSS group. 7-KDCA, 7-ketodeoxycholic acid; DSS, dextran sulfate sodium; TNF-α, tumour necrosis factor alpha; IL-6, interleukin 6; IL-1β, interleukin-1beta; TFF3, trefoil factor 3; TGF-β, transforming growth factor-beta; qPCR, quantitative real-time polymerase chain reaction.

**
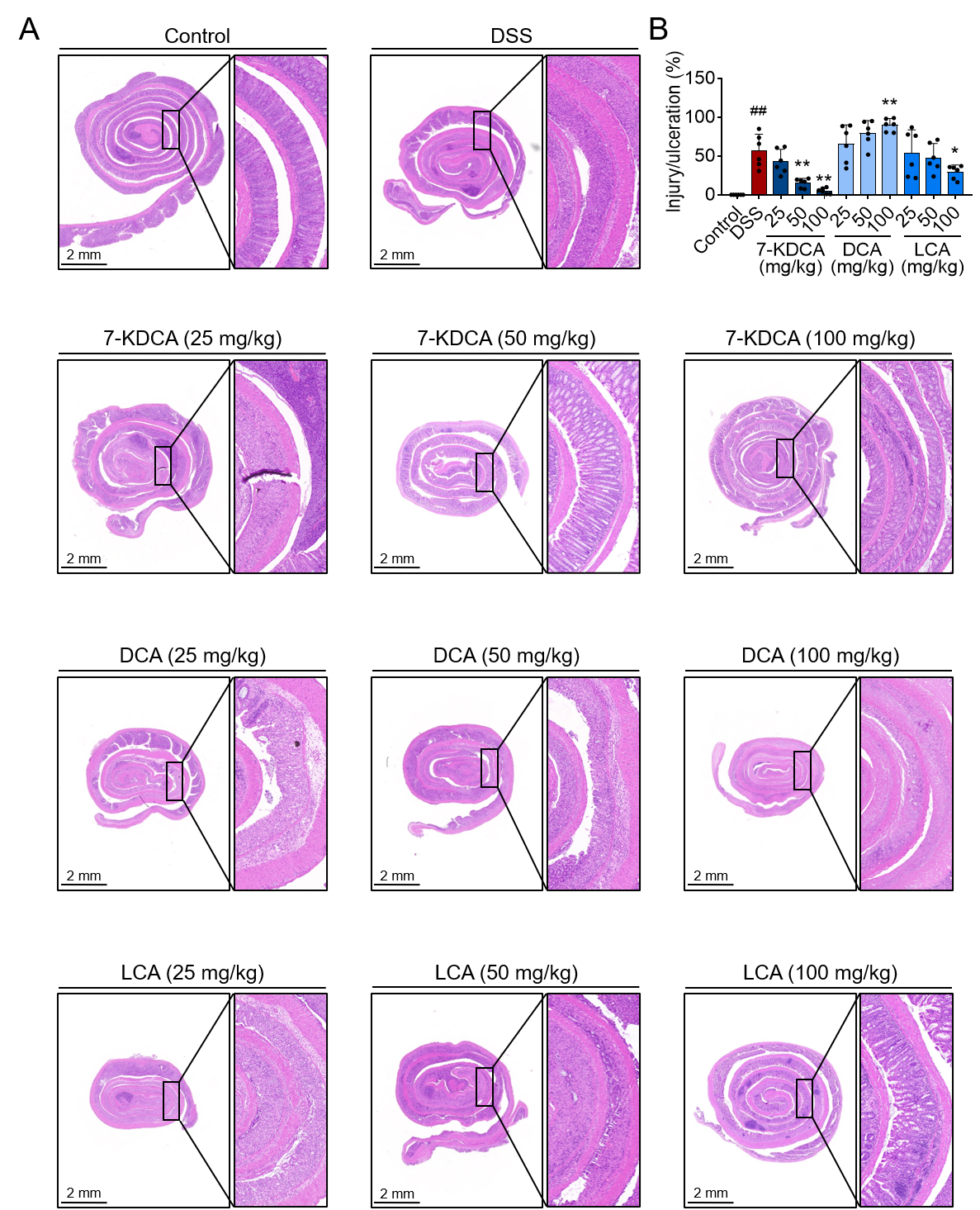
**

**Figure S7.** 7-KDCA facilitates colonic mucosal wound healing in mice with DSS-induced colitis. C57BL/6 mice underwent a 5-day exposure to 3.5% DSS, succeeded by a subsequent 5 days of water together with vehicle or bile acids. (A) Representative images of Swiss roll colon sections stained with H&E. The amplified details correspond to the black regions within the insets. (B) Histological colitis score denotes a ratio of the length of injured/ulcerated areas in relation to the overall length of the colon. This evaluation is conducted through Swiss roll mounts encompassing the entire colon. Data represent mean ± SD (n = 6). ^##^P < 0.01 vs. Control group. *P < 0.05, **P < 0.01 vs. DSS group. 7-KDCA, 7-ketodeoxycholic acid; DSS, dextran sulfate sodium; H&E, hematoxylin and eosin.

**
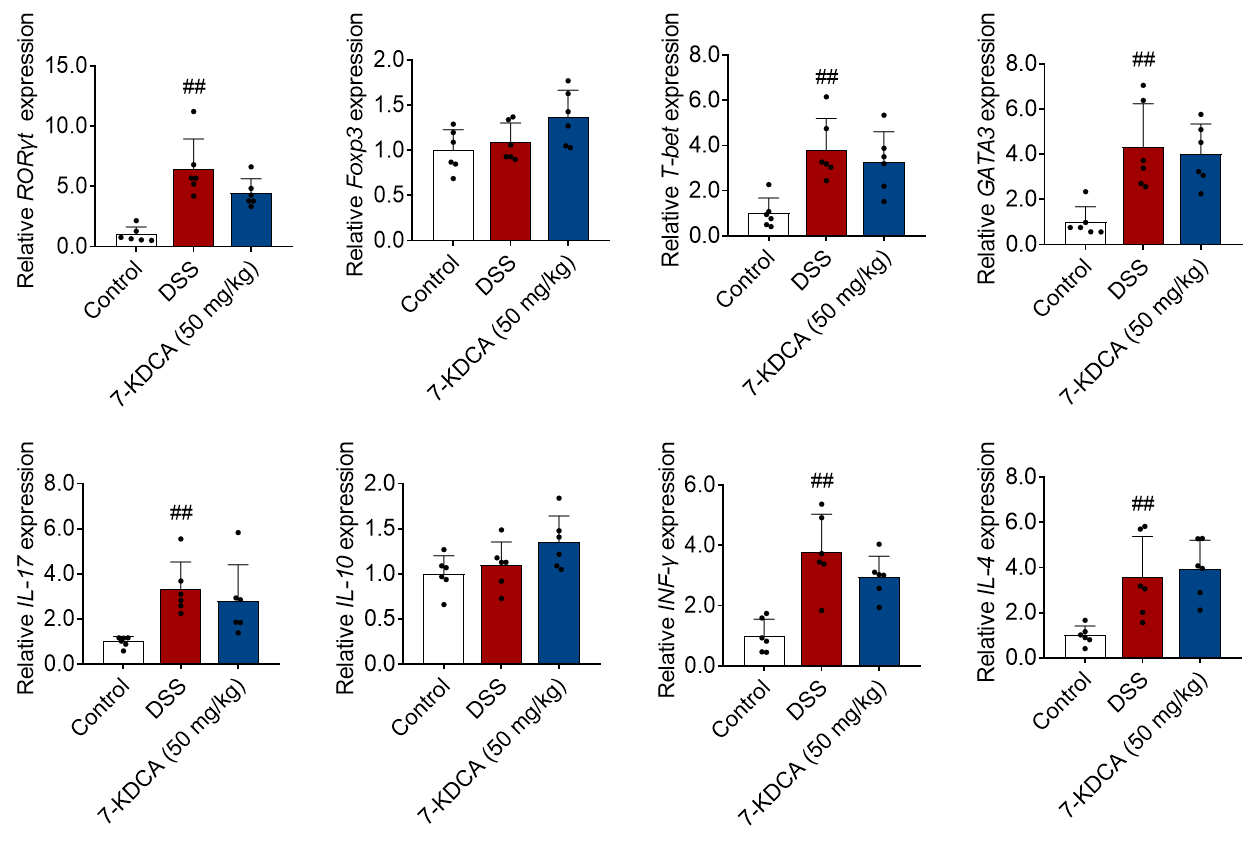
**

**Figure S8.** Effect of 7-ketodeoxycholic acid on the mRNA expression of RORγt, Foxp3, T-bet, GATA3, IL-17, IL-10, INF-γ and IL-4 in colons of mice with DSS-induced colitis. C57BL/6 mice underwent a 5-day exposure to 3.5% DSS, succeeded by a subsequent 5 days of water together with vehicle or 7-ketodeoxycholic acid. Data represent mean ± SD (n = 6). ^##^P < 0.01 vs. Control group. 7-KDCA, 7-ketodeoxycholic acid; DSS, dextran sulfate sodium.

**
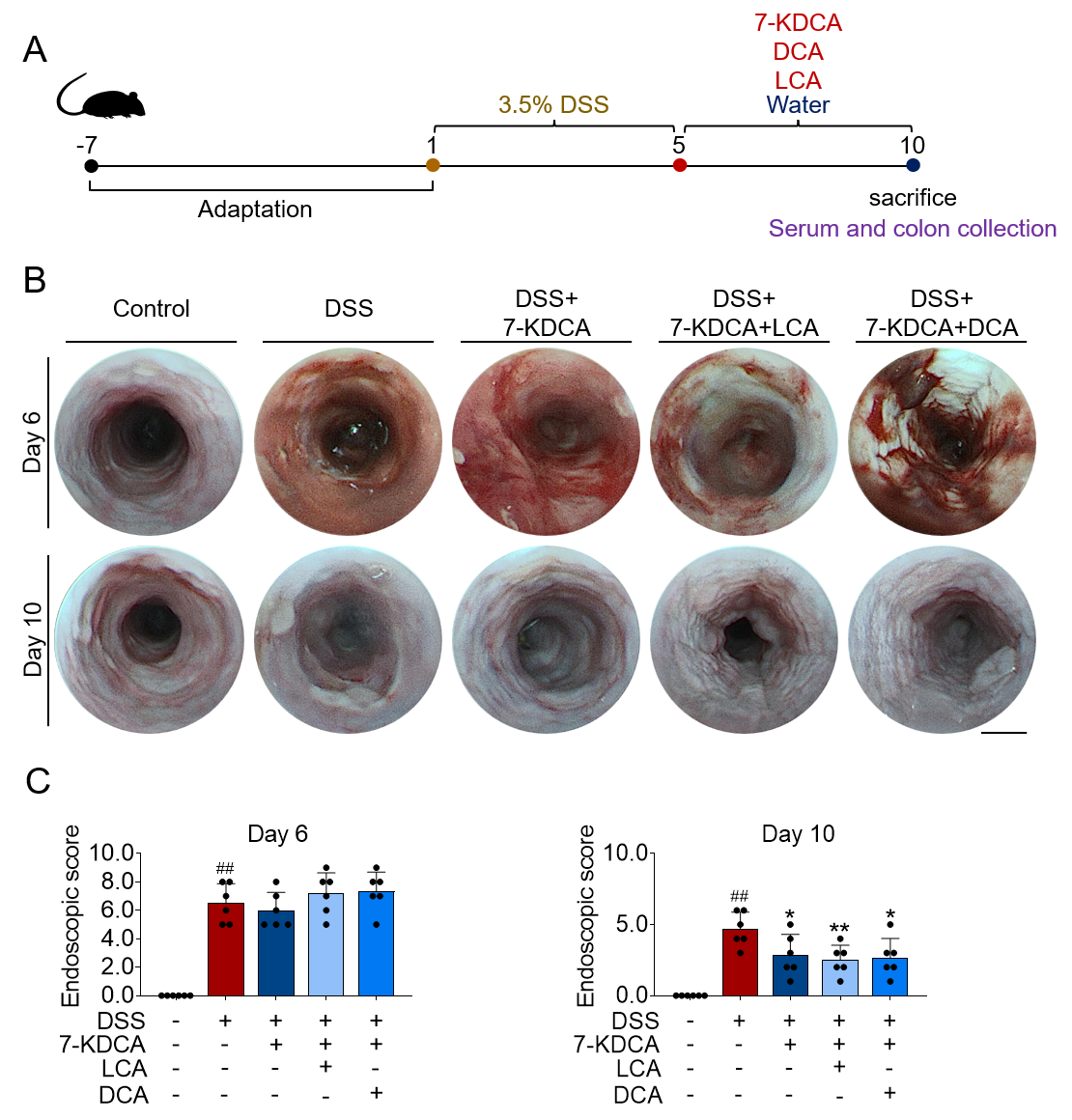
**

**Figure S9.** The function of 7-KDCA in promoting colonic mucosal healing remains unaffected in the presence of other bile acids. C57BL/6 mice underwent a 5-day exposure to 3.5% DSS, succeeded by a subsequent 5 days of water together with vehicle or bile acids. (A) Strategy for bile acids treatment during the recovery period of DSS colitis model. (B) Representative images displaying regions characterized by overt bleeding and edema. (C) Endoscopic score. Data represent mean ± SD (n = 6). ^##^P < 0.01 vs. Control group. *P < 0.05, **P < 0.01 vs. DSS group. 7-KDCA, 7-ketodeoxycholic acid; DSS, dextran sulfate sodium; DCA, deoxycholic acid; LCA, lithocholic acid.

**
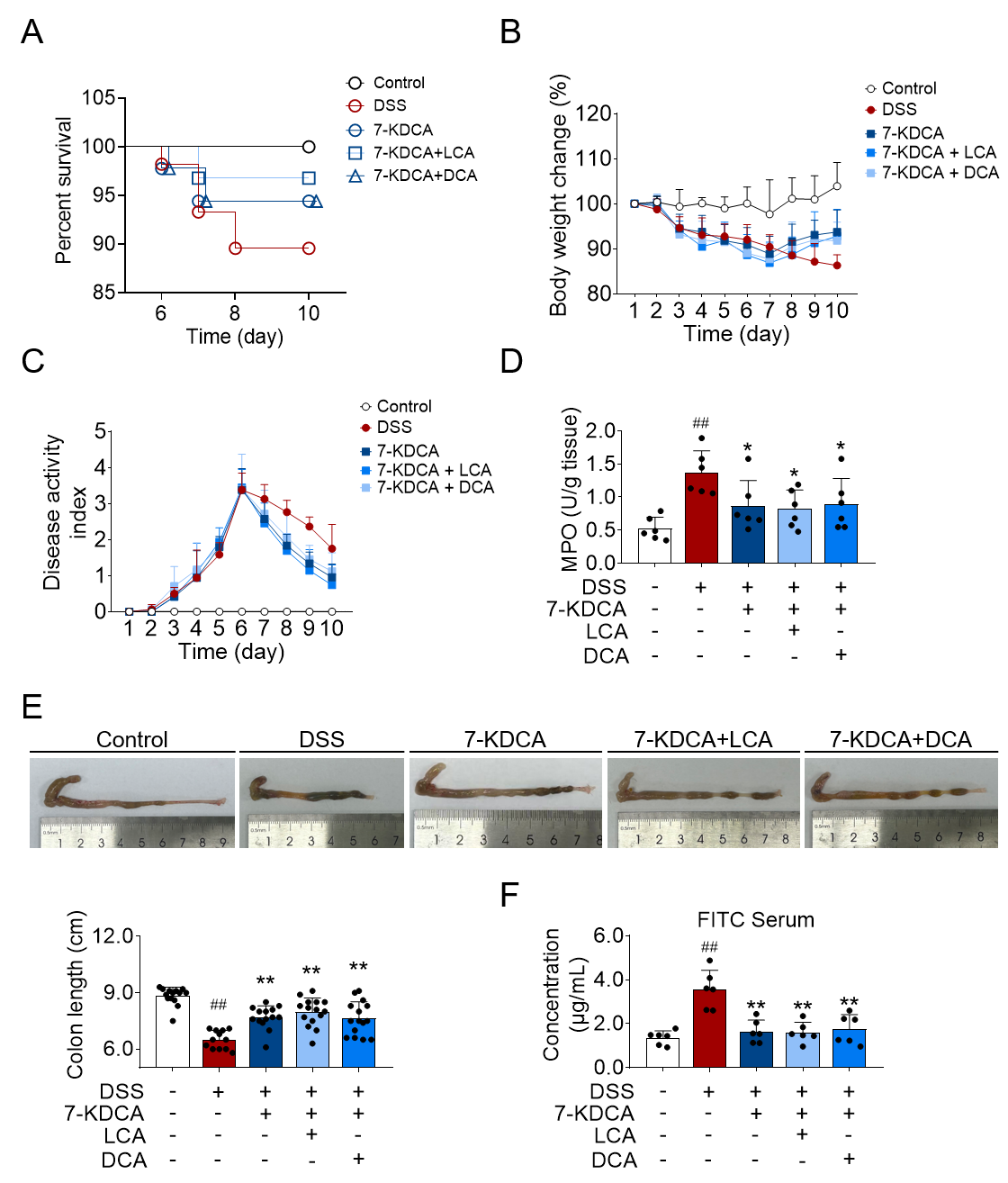
**

**Figure S10.** The function of 7-KDCA in ameliorating experimental colitis remains unaffected in the presence of other bile acids. C57BL/6 mice underwent a 5-day exposure to 3.5% DSS, succeeded by a subsequent 5 days of water together with vehicle or bile acids. (A) Survival rate (d, days). (B) Percentage change in body weight. (C) DAI score. (D) MPO activity in colon tissues. (E) Colon length. (F) Relative fluorescence intensity of FITC-dextran in serum. Data represent mean ± SD (n = 14-16). ^##^P < 0.01 vs. Control group. *P < 0.05, **P < 0.01 vs. DSS group. 7-KDCA, 7-ketodeoxycholic acid; DSS, dextran sulfate sodium; DCA, deoxycholic acid; LCA, lithocholic acid; DAI, disease activity index; MPO, myeloperoxidase; FITC, fluorescein isothiocyanate.

**
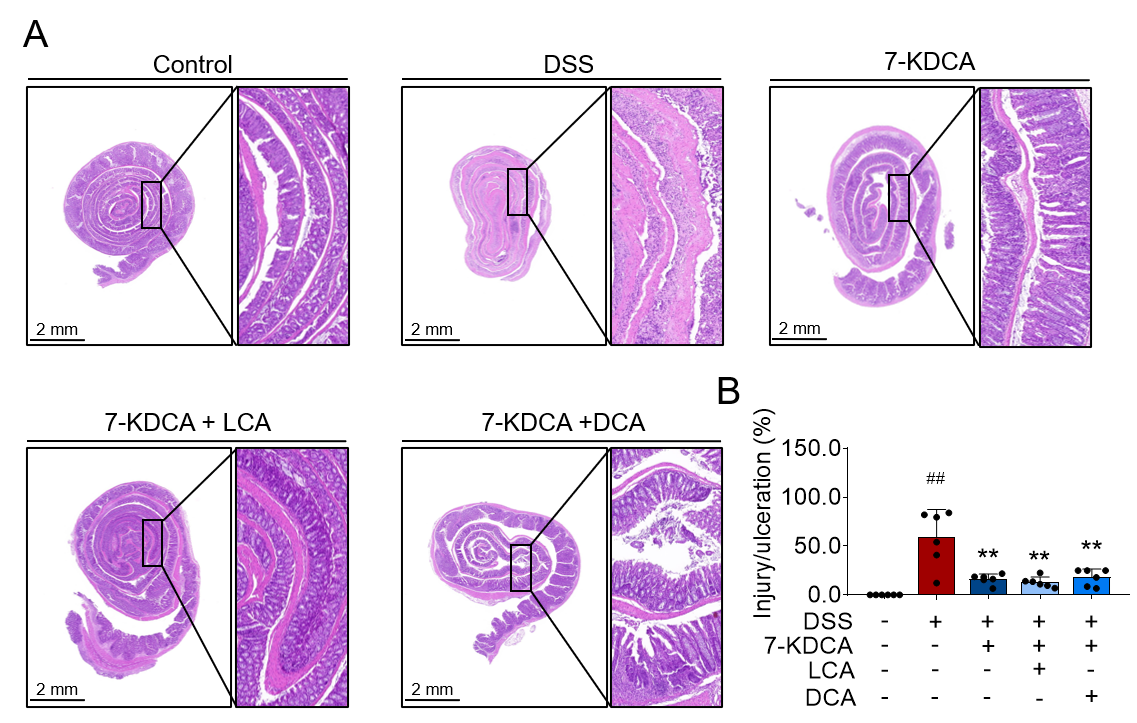
**

**Figure S11.** The function of 7-KDCA in promoting colonic mucosal healing remains unaffected in the presence of other bile acids. C57BL/6 mice underwent a 5-day exposure to 3.5% DSS, succeeded by a subsequent 5 days of water together with vehicle or bile acids. (A) Representative images of Swiss roll colon sections stained with H&E. The amplified details correspond to the black regions within the insets. (B) Histological colitis score denotes a ratio of the length of injured/ulcerated areas in relation to the overall length of the colon. This evaluation is conducted through Swiss roll mounts encompassing the entire colon. Data represent mean ± SD (n = 6). ^##^P < 0.01 vs. Control group. **P < 0.01 vs. DSS group. 7-KDCA, 7-ketodeoxycholic acid; DSS, dextran sulfate sodium; DCA, deoxycholic acid; LCA, lithocholic acid; H&E, hematoxylin and eosin.

**
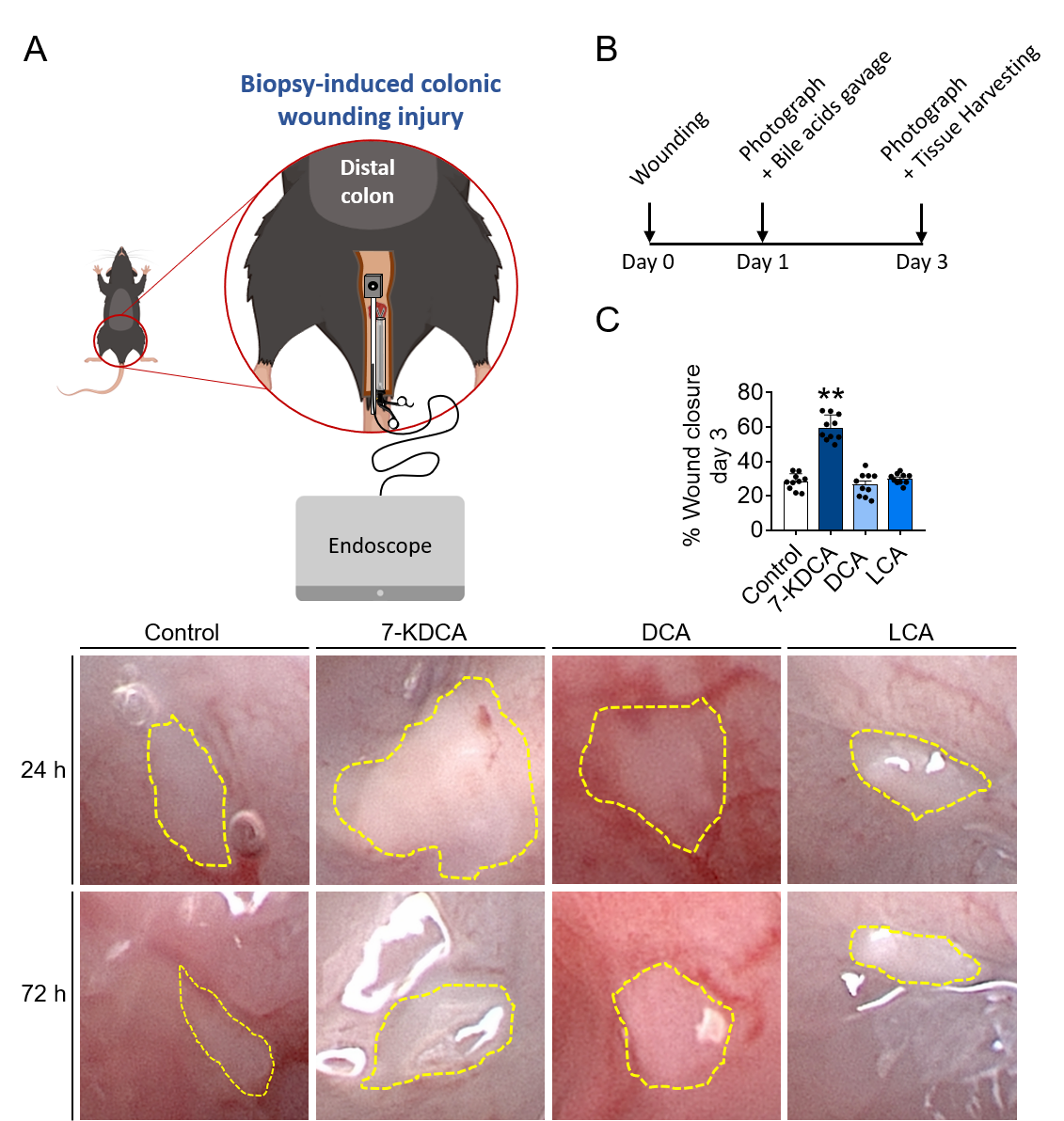
**

**Figure S12.** 7-KDCA promotes the restoration of colonic wounds induced by biopsy *in vivo*. (A) Lesions were induced on the dorsal aspect of the distal colon. (B) Schematic overview of experimental design timeline. Employing a diminutive video endoscope and biopsy scissors, lesions were generated within the dorsal aspect of the mucosa in the descending colon of anesthetized mice on day 0. Suspension of 7-KDCA or DCA or LCA or vehicle control was intragastrically given 24 h post-wounding. (C) Digital photos of the wound surface area were taken at 24 and 72 h post-injury. The periphery of the wounds was delineated by yellow dashed lines. The assessment of colonic mucosal wound healing involved the measurement of wound areas at 24 and 72 h, subsequently normalized against the vehicle control. Data represent mean ± SD (n = 8). **P < 0.01 vs. Vehicle control group. 7-KDCA, 7-ketodeoxycholic acid; DCA, deoxycholic acid; LCA, lithocholic acid.

**
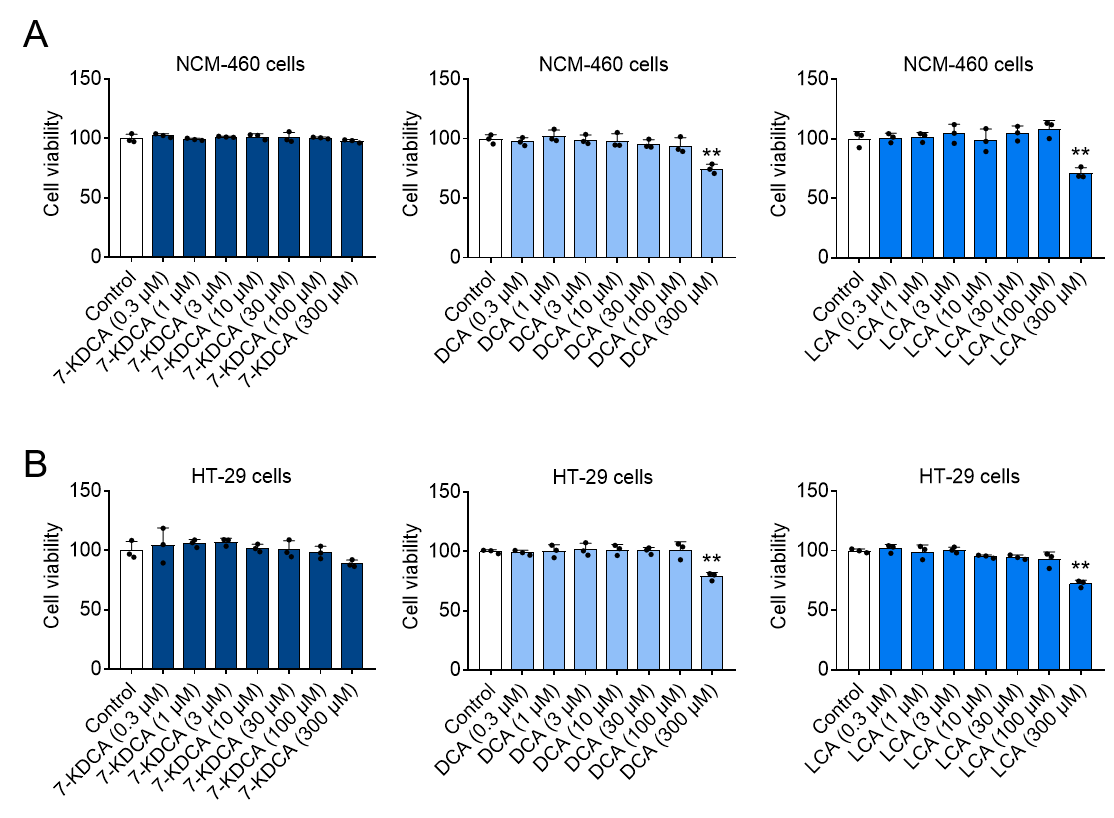
**

**Figure S13.** 7-KDCA does not exhibit toxicity towards human intestinal cells at physiologically relevant concentrations. Colonic epithelial cells were cultured in the presence or absence of 7-KDCA or DCA or LCA. Viability of (A) NCM-460 cells and (B) HT-29 cells was assessed through MTT assay after 24 h. Data represent mean ± SD (n = 3). **P < 0.01 vs. Control group. 7-KDCA, 7-ketodeoxycholic acid; DCA, deoxycholic acid; LCA, lithocholic acid; MTT, 3-(4, 5-dimethylthiazol-2-yl)-2, 5-diphenyl tetrazolium bromide.

**
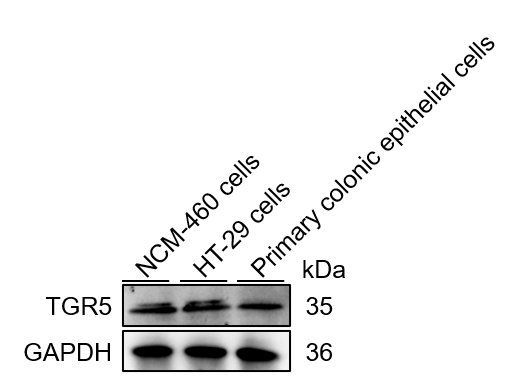
**

**Figure S14.** Western blot analysis of TGR5 in NCM-460, HT-29 cells, and primary colonic epithelial cells. Data represent mean ± SD (n = 3). TGR5, Takeda G protein-coupled receptor 5.

**
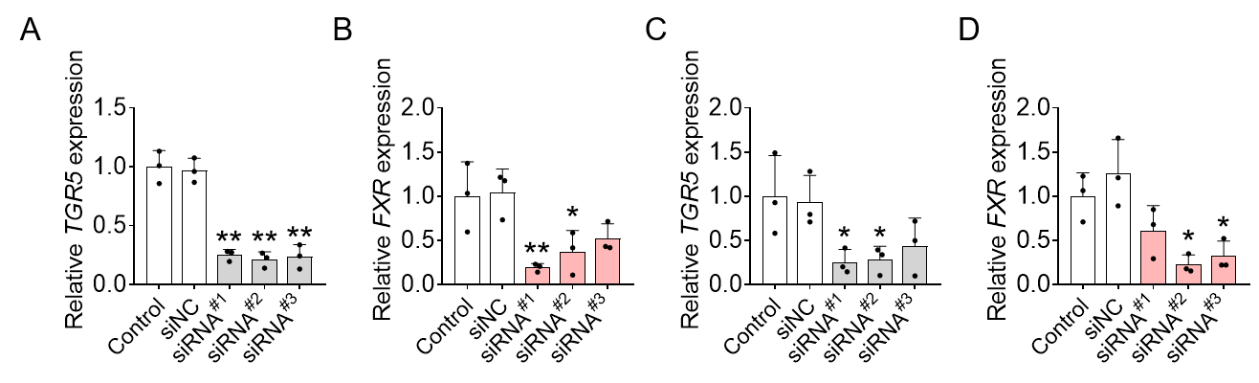
**

**Figure S15.** Validation of the knockdown efficiency of TGR5 and FXR using small interfering RNA in human colonic epithelial cells. (A) qPCR analysis of *TGR5* expression in NCM-460 cells transfected with/without siNC or siTGR5 for 24 h. (B) qPCR analysis of *FXR* expression in NCM-460 cells transfected with/without siNC or siFXR for 24 h. (C) qPCR analysis of *TGR5* expression in primary colonic epithelial cells transfected with/without siNC or siTGR5 for 24 h. (D) qPCR analysis of *FXR* expression in primary colonic epithelial cells transfected with/without siNC or siFXR for 24 h. Data represent mean ± SD (n = 3). *P < 0.05, **P < 0.01 vs. siNC group. siNC, negative control siRNA; siTGR5, small interfering RNA of TGR5; siFXR, small interfering RNA of FXR.

**
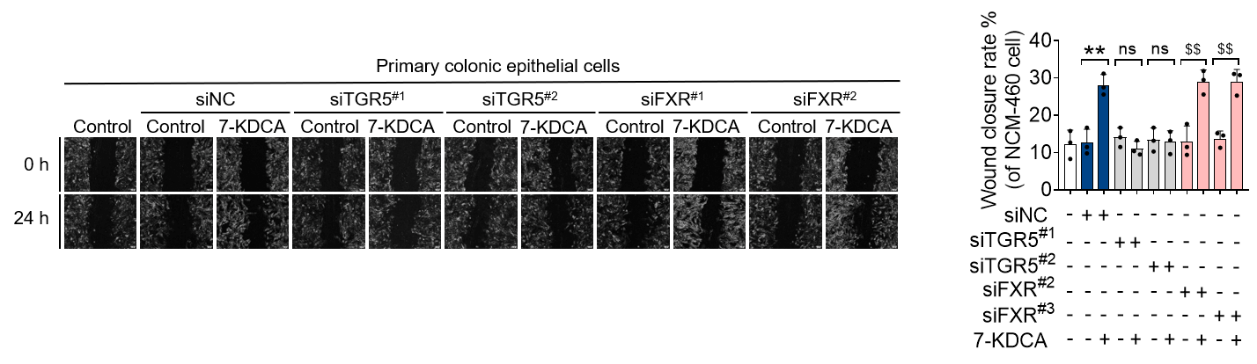
**

**Figure S16.** Primary colonic epithelial cells were transfected with siNC or siTGR5 or siFXR, treated with/without 7-KDCA (10 μM) for 24 h, and subjected to wound closure assay (illustrated through a representative scratch assay). Data represent mean ± SD (n = 3). **P < 0.01 vs. siNC group. 7-KDCA, 7-ketodeoxycholic acid; siNC, negative control siRNA; siTGR5, small interfering RNA of TGR5; siFXR, small interfering RNA of FXR.

**
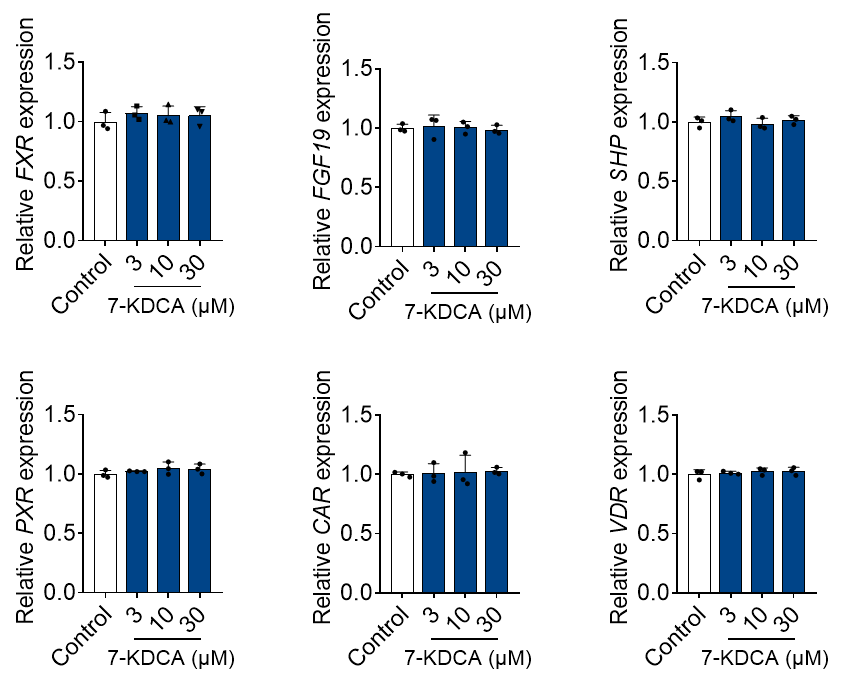
**

**Figure S17.** 7-KDCA exhibits no discernible impact on the expression levels of *FXR*, *FGF19*, *SHP*, *PXR*, *CAR* and *VDR* in NCM-460 cells at tested concentrations. The mRNA expression of *FXR*, *FGF19*, *SHP*, *PXR*, *CAR* and *VDR* in NCM-460 cells after 7-KDCA intervention. Data represent mean ± SD (n = 3). 7-KDCA, 7-ketodeoxycholic acid; FXR, farnesoid X receptor; FGF19, fibroblast growth factor 19; SHP, small heterodimer partner; PXR, pregnane X receptor; CAR, constitutive androstane receptor; VDR, vitamin D receptor.

**
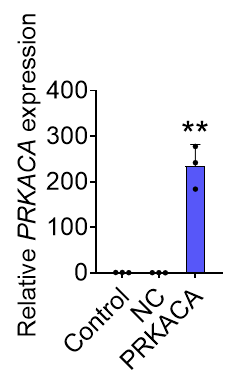
**

**Figure S18.** qPCR analysis of *PRKACA* expression in NCM-460 cells with/without PRKACA overexpression for 24 h. Data represent mean ± SD (n = 3). **P < 0.01 vs. NC group. qPCR, quantitative real-time polymerase chain reaction; NC, normal control.

**
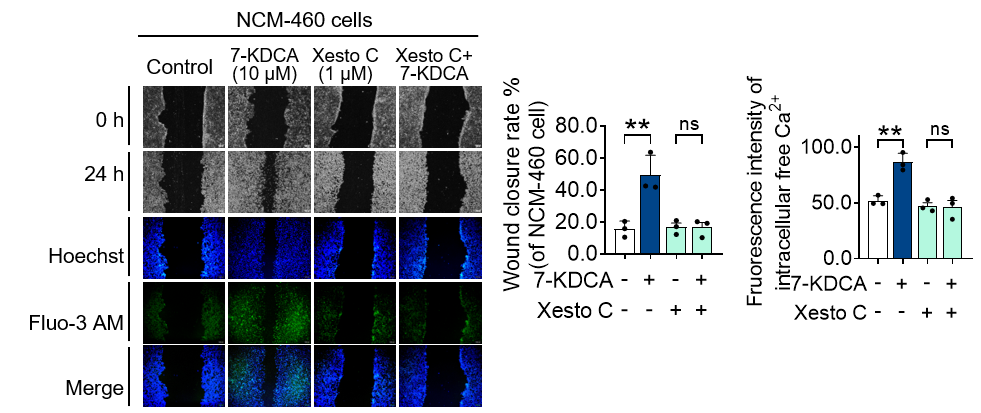
**

**Figure S19.** NCM-460 cells were treated with/without 7-KDCA in the presence or absence of 1 μM xestospongin C for 24 h, and subjected to wound closure (illustrated through a representative scratch assay) and analysis of cytoplasmic Ca^2+^ level. Data represent mean ± SD (n = 3). **P < 0.01 vs. Control group. ns, no significant difference. 7-KDCA, 7-ketodeoxycholic acid; Xesto C, xestospongin C.

**
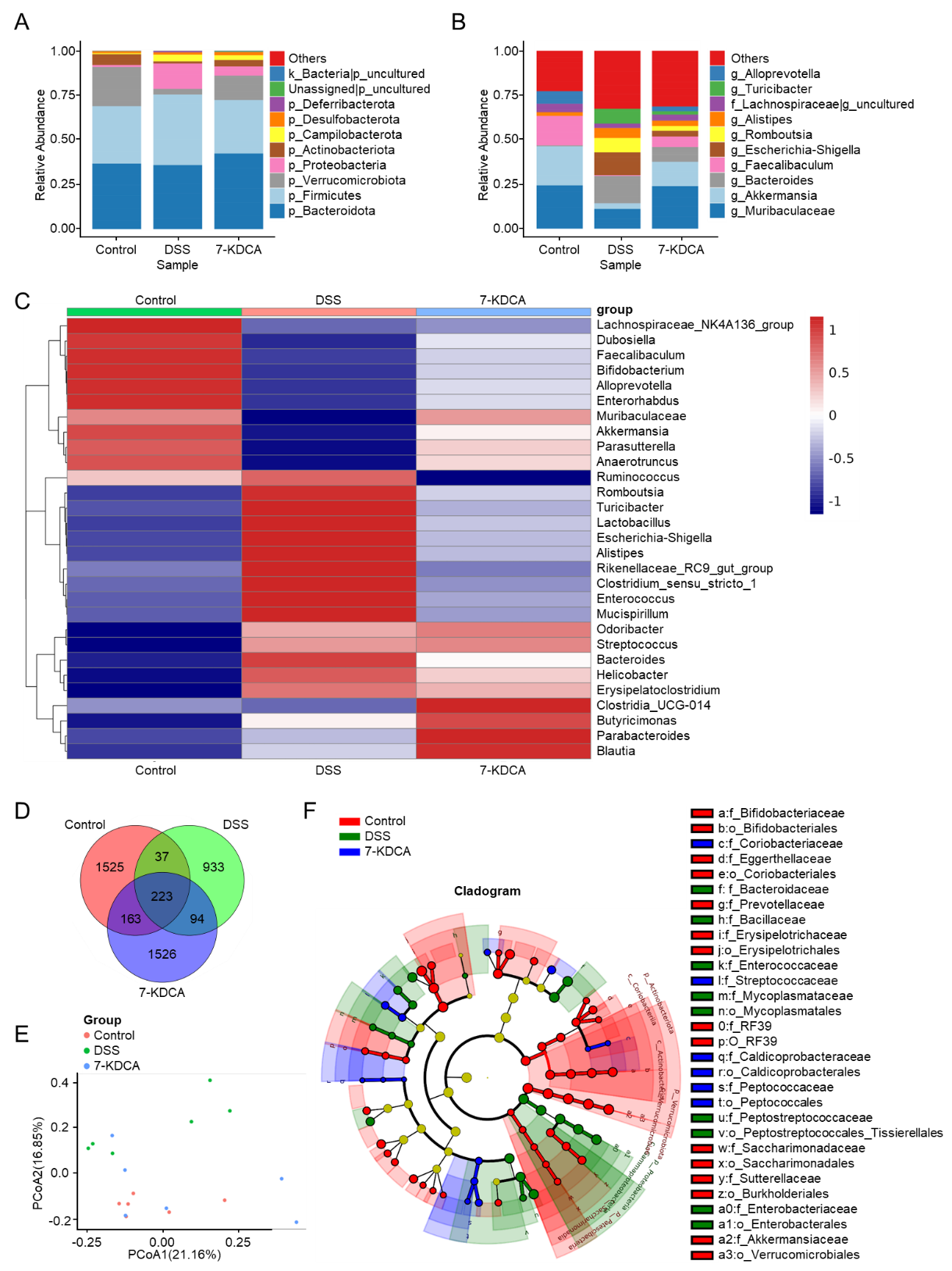
**

**Figure S20** Effect of 7-ketodeoxycholic acid on the composition of intestinal flora in mice with DSS-induced colitis. The percent of community abundance at the phylum (A) and genus (B) levels. (C) Heat map showing the species composition for species clustering. (D) OUT Venn diagram. (E) PCoA diagram, each point in the diagram represents a sample. (F) A diagram illustrating the inter-group difference classification units based on classification hierarchy tree.


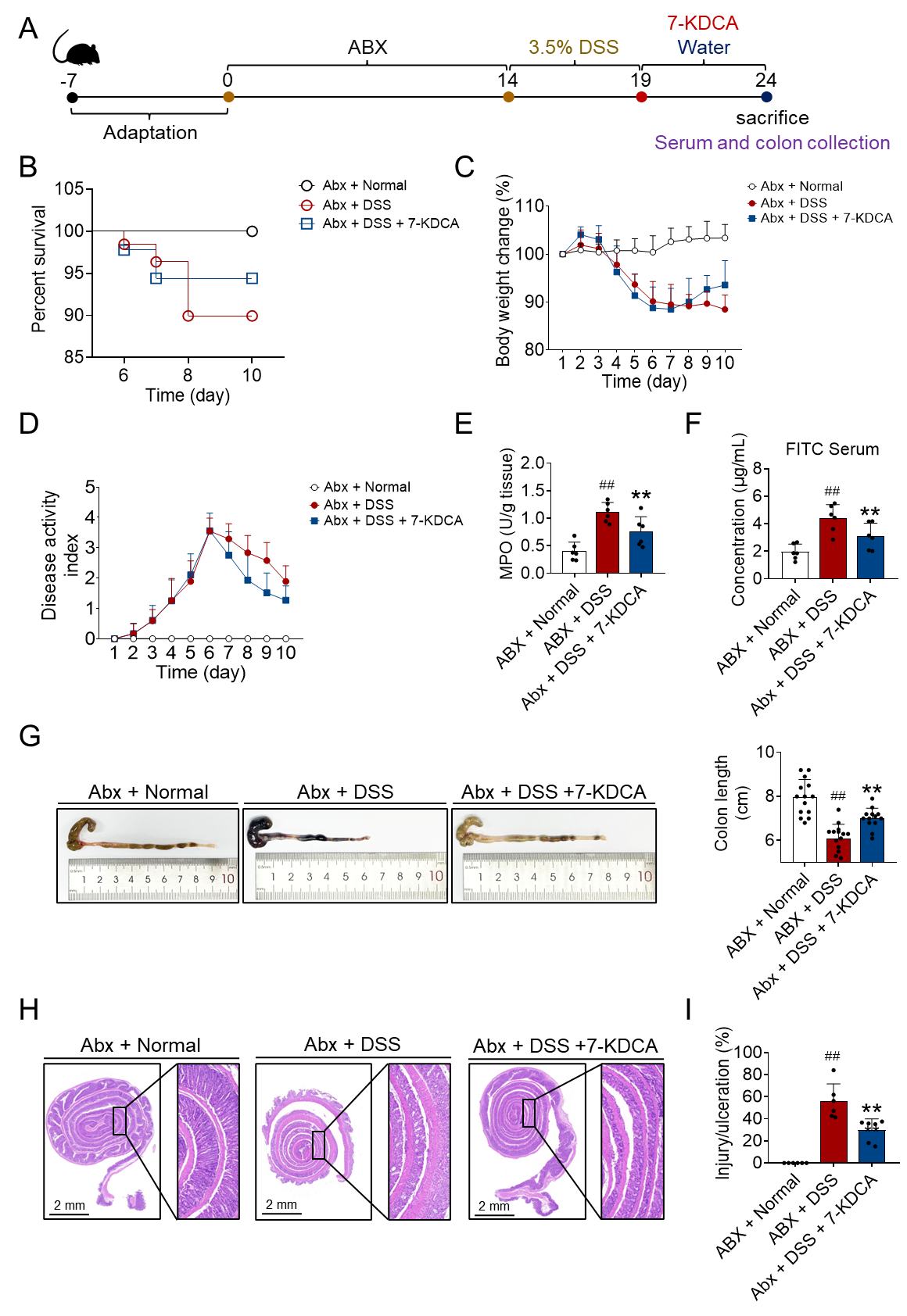


**Figure S21.** The facilitation of colonic mucosal wound healing and anti-colitis effect of 7-ketodeoxycholic acid are independent of the gut microbiota. (A) Experimental design. (B) Survival rate (d, days). (C) Percentage change in body weight. (D) DAI score. (E) MPO activity in colon tissues. (F) Relative fluorescence intensity of FITC-dextran in serum. (G) Colon length. (H) Representative images of Swiss roll colon sections stained with H&E. The amplified details correspond to the black regions within the insets. (I) Histological colitis score denotes a ratio of the length of injured/ulcerated areas in relation to the overall length of the colon. This evaluation is conducted through Swiss roll mounts encompassing the entire colon. Data represent mean ± SD (n = 13-15). ^##^P < 0.01 vs. Abx + Normal group. **P < 0.01 vs. Abx + DSS group. 7-KDCA, 7-ketodeoxycholic acid; H&E, hematoxylin and eosin; DSS, dextran sulfate sodium; Abx, antibiotic.

**
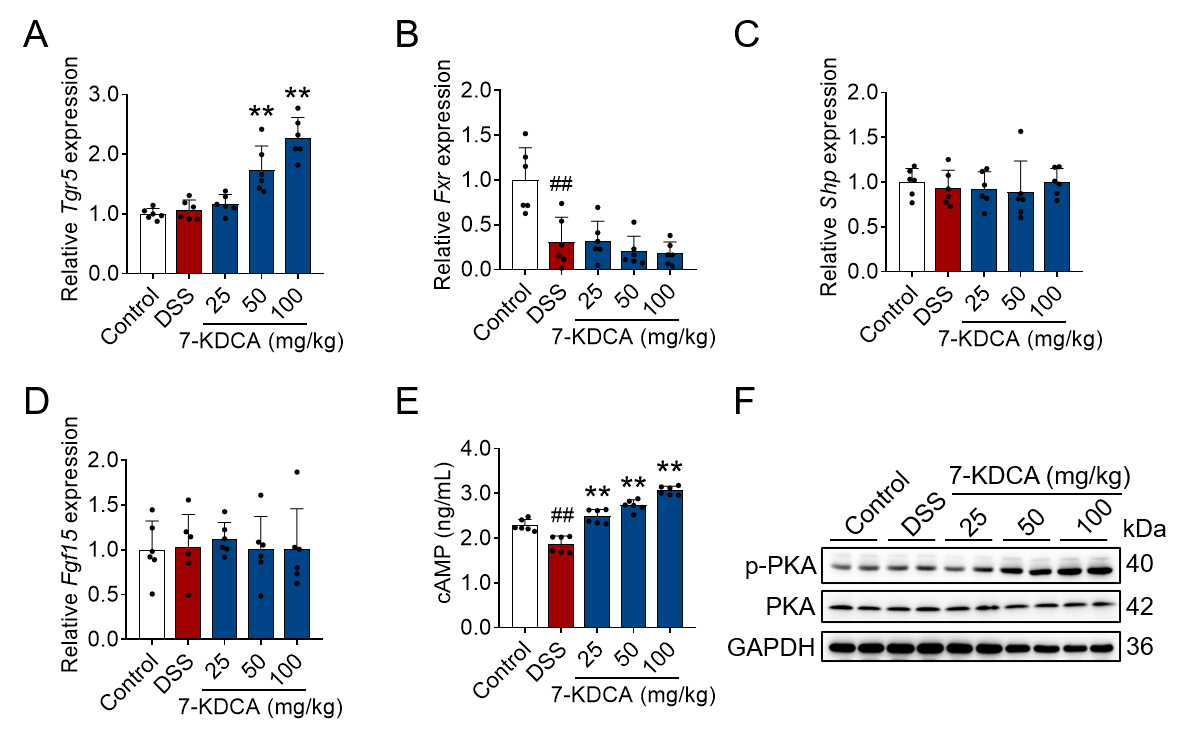
**

**Figure S22.** 7-KDCA activates TGR5 signaling and increases *Tgr5* expression in colon tissues in mice with DSS-induced colitis. (A-D) The mRNA levels of *Tgr5*, *Fxr*, *Shp* and *Fgf15* in colon tissues as determined by qPCR assay. (E) cAMP level in colon tissues was measured by ELISA. (F) Western blot analysis of PKA and p-PKA protein levels in colon tissues. Data represent mean ± SD (n = 6). ^##^P < 0.01 vs. Control group. **P < 0.01 vs. DSS group. 7-KDCA, 7-ketodeoxycholic acid; TGR5, Takeda G protein-coupled receptor 5; DSS, dextran sulfate sodium; FXR, farnesoid X receptor; SHP, small heterodimer partner; FGF15, fibroblast growth factor 15; qPCR, quantitative real-time polymerase chain reaction; cAMP, cyclic adenosine monophosphate; ELISA, enzyme-linked immunosorbent assay; PKA, protein kinase A; p-PKA, phospho-PKA.

**
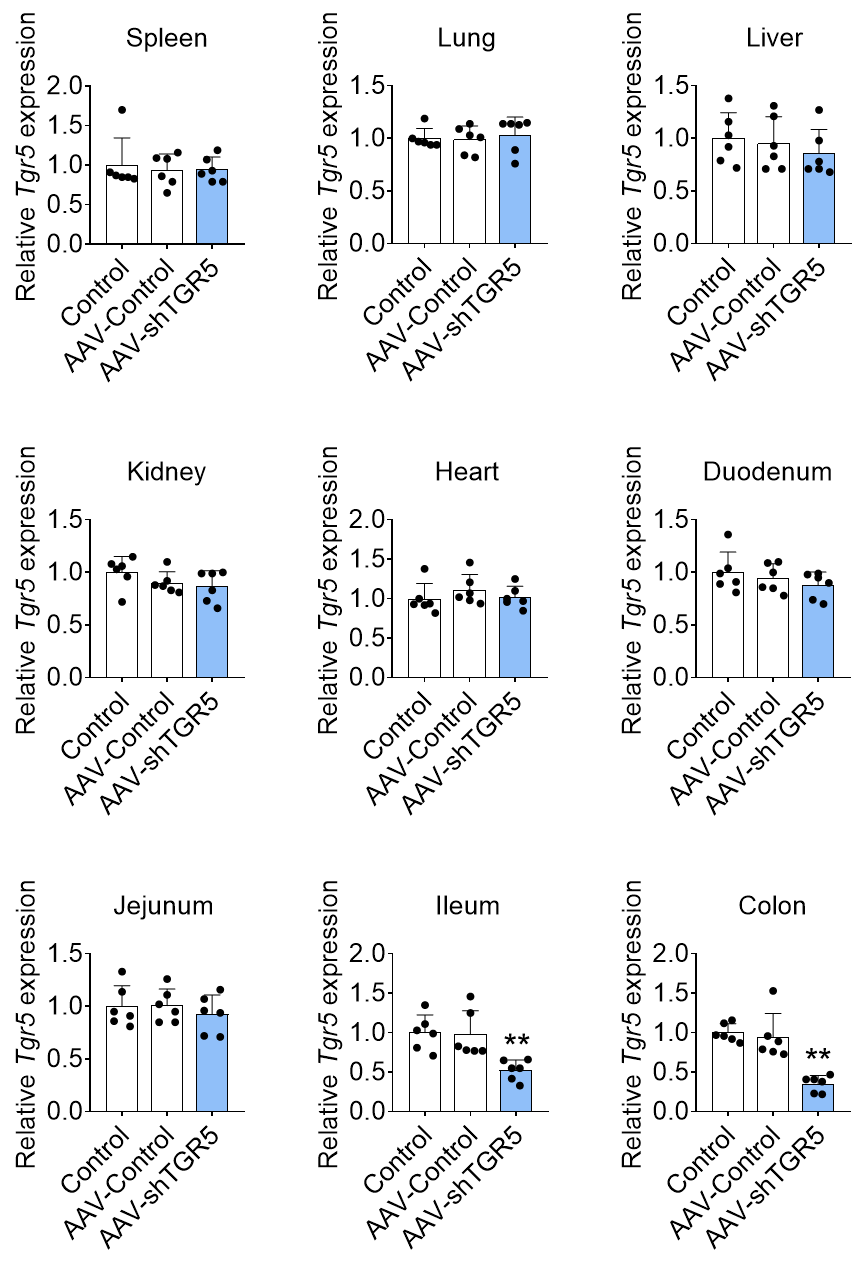
**

**Figure S23.** The expression of *Tgr5* in the ileum and colon was dramatically decreased by AAV-TGR5-shRNA enema, but the expression of *Tgr5* in spleen, lungs, liver, kidneys, heart, duodenum and jejunum was not significantly altered. Data represent mean ± SD (n = 6). **P < 0.01 vs. AAV-Control group. TGR5, Takeda G protein-coupled receptor 5.

**
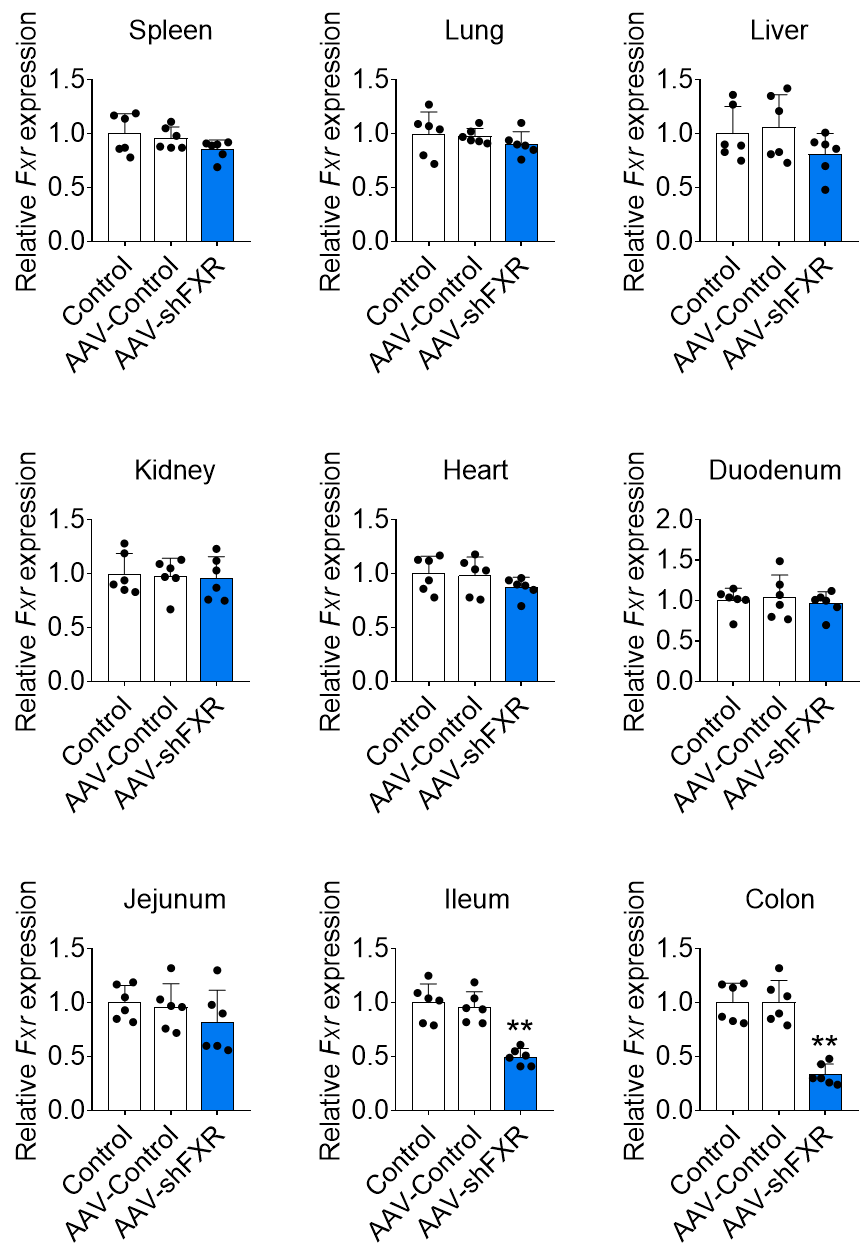
**

**Figure S24.** The expression of *Fxr* in the ileum and colon was dramatically decreased by AAV-FXR-shRNA enema, but the expression of *Fxr* in spleen, lungs, liver, kidneys, heart, duodenum and jejunum was not significantly altered. Data represent mean ± SD (n = 6). **P < 0.01 vs. AAV-Control group. FXR, farnesoid X receptor.

**
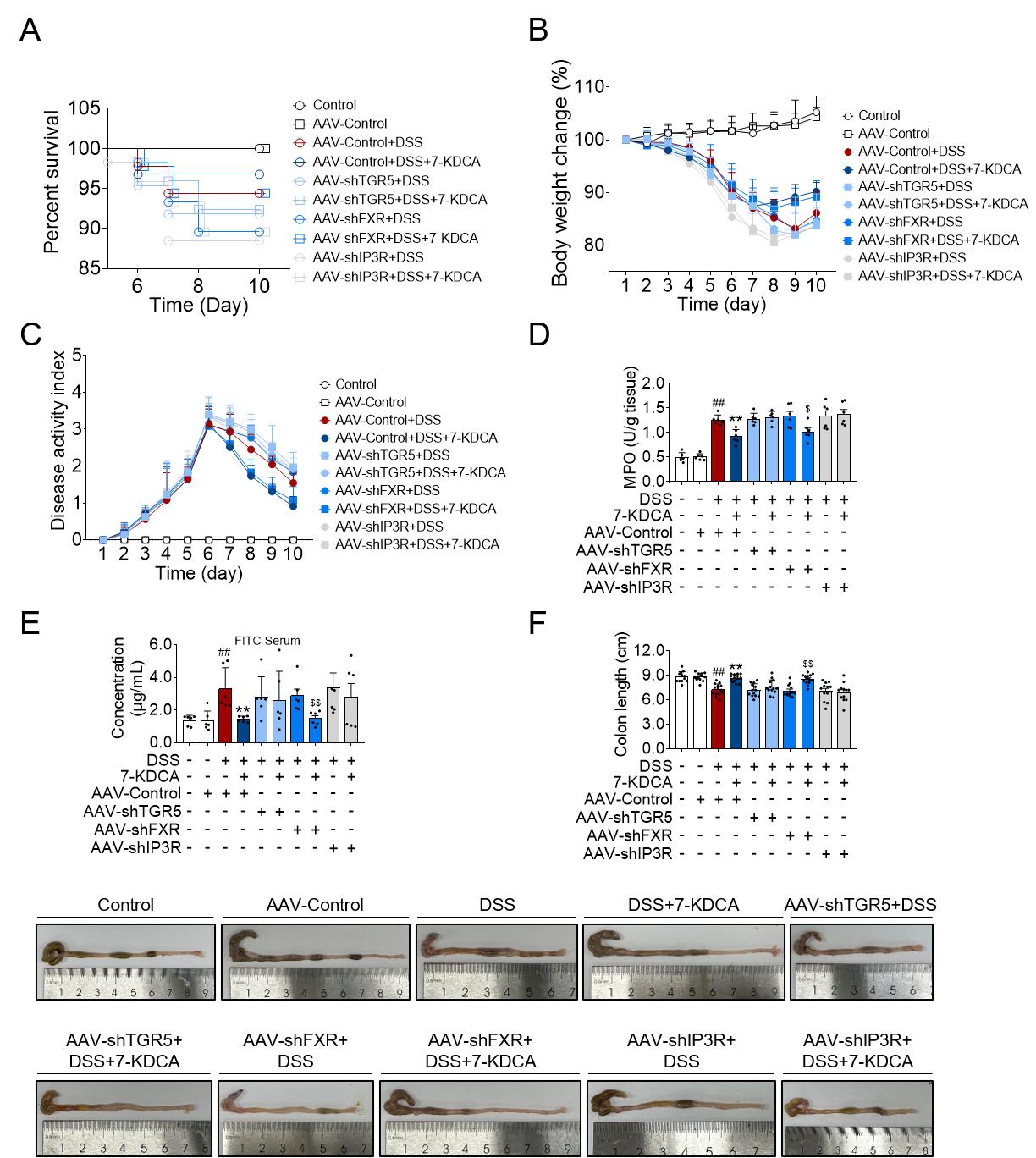
**

**Figure S25.** 7-KDCA mitigates colitis by targeting colonic TGR5-IP3R signaling. Comparison of biochemical parameters of mice among multiple groups. (A) Survival rate (d, days). (B) Percentage change in body weight. (C) DAI score. (D) MPO activity in colon tissues. (E) Relative fluorescence intensity of FITC-dextran in serum. (F) Colon length. Data represent mean ± SD (n = 14-16). ^##^P < 0.01 vs. AAV-Control group. **P < 0.01 vs. DSS group. ^$^P < 0.05, ^$$^P < 0.01 vs. AAV-shFXR group. 7-KDCA, 7-ketodeoxycholic acid; TGR5, Takeda G protein-coupled receptor 5; FXR, farnesoid X receptor; IP3R, inositol 1,4,5-trisphosphate receptor; DSS, dextran sulfate sodium; DAI, disease activity index; MPO, myeloperoxidase; FITC, fluorescein isothiocyanate.

**
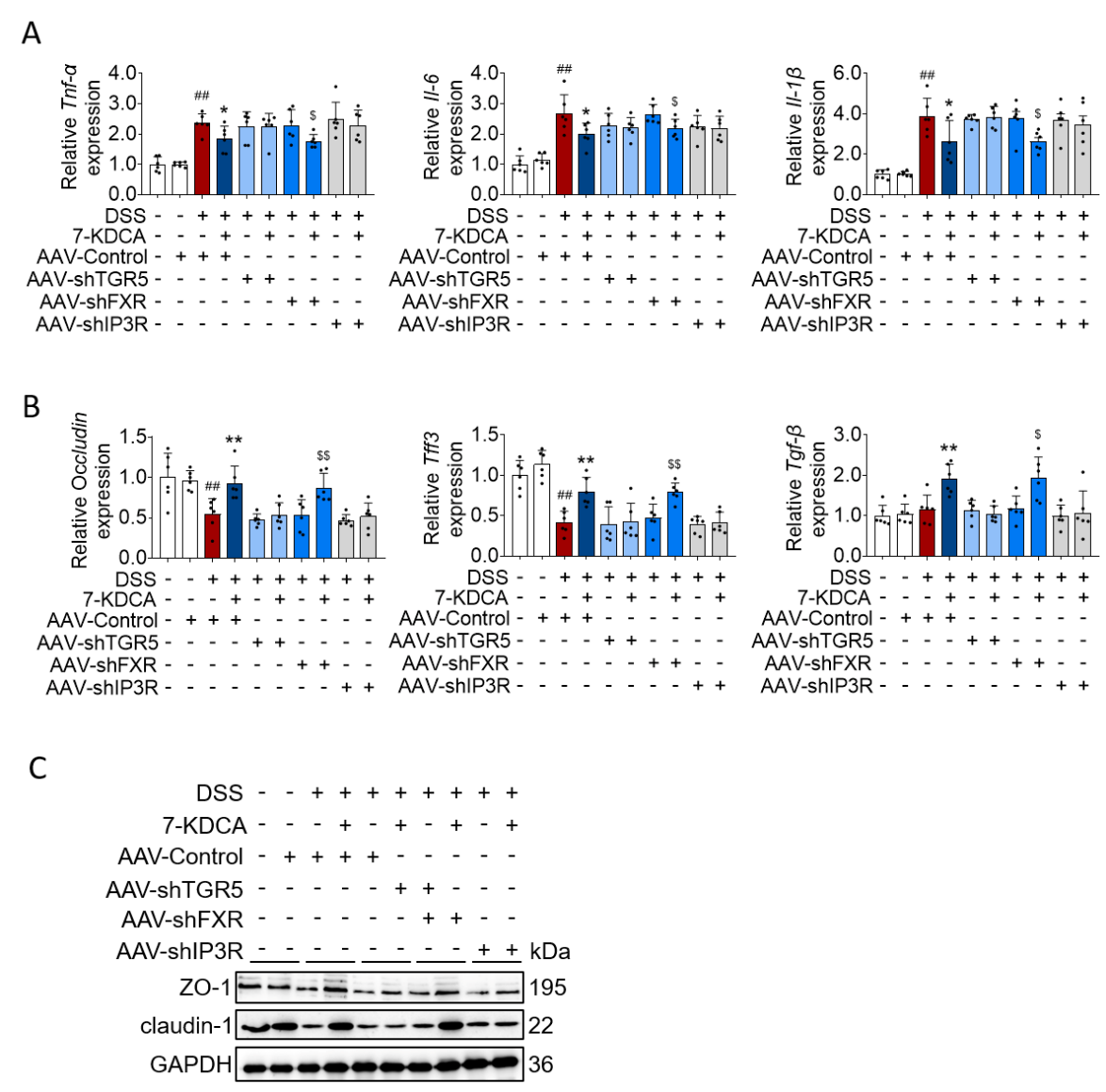
**

**Figure S26.** 7-KDCA adjusts the levels of proinflammatory cytokines factors and cytokines associated with mucosal repair in FXR knockdown mice, while 7-KDCA fails to modulate the expression of these genes in colitis mice lacking TGR5 or IP3R. (A) The mRNA expression of *Tnf-α*, *Il-6* and *Il-1β* in colon tissues as determined by qPCR assay. (B) The mRNA expression of *Occludin*, *Tff3* and *Tgf-β* in colon tissues as determined by qPCR assay. (C) Western blot analysis of ZO-1 and claudin-1 protein levels in colon tissues. Data represent mean ± SD (n = 6). ^##^P < 0.01 vs. Control group. *P < 0.05, **P < 0.01 vs. DSS group. ^$^P < 0.05, ^$$^P < 0.01 vs. AAV-shFXR group. 7-KDCA, 7-ketodeoxycholic acid; FXR, farnesoid X receptor; TGR5, Takeda G protein-coupled receptor 5; IP3R, inositol 1,4,5-triphosphate receptor; DSS, dextran sulfate sodium; TNF-α, tumour necrosis factor alpha; IL-6, interleukin 6; IL-1β, interleukin-1beta; TFF3, trefoil factor 3; TGF-β, transforming growth factor-beta; qPCR, quantitative real-time polymerase chain reaction.

**
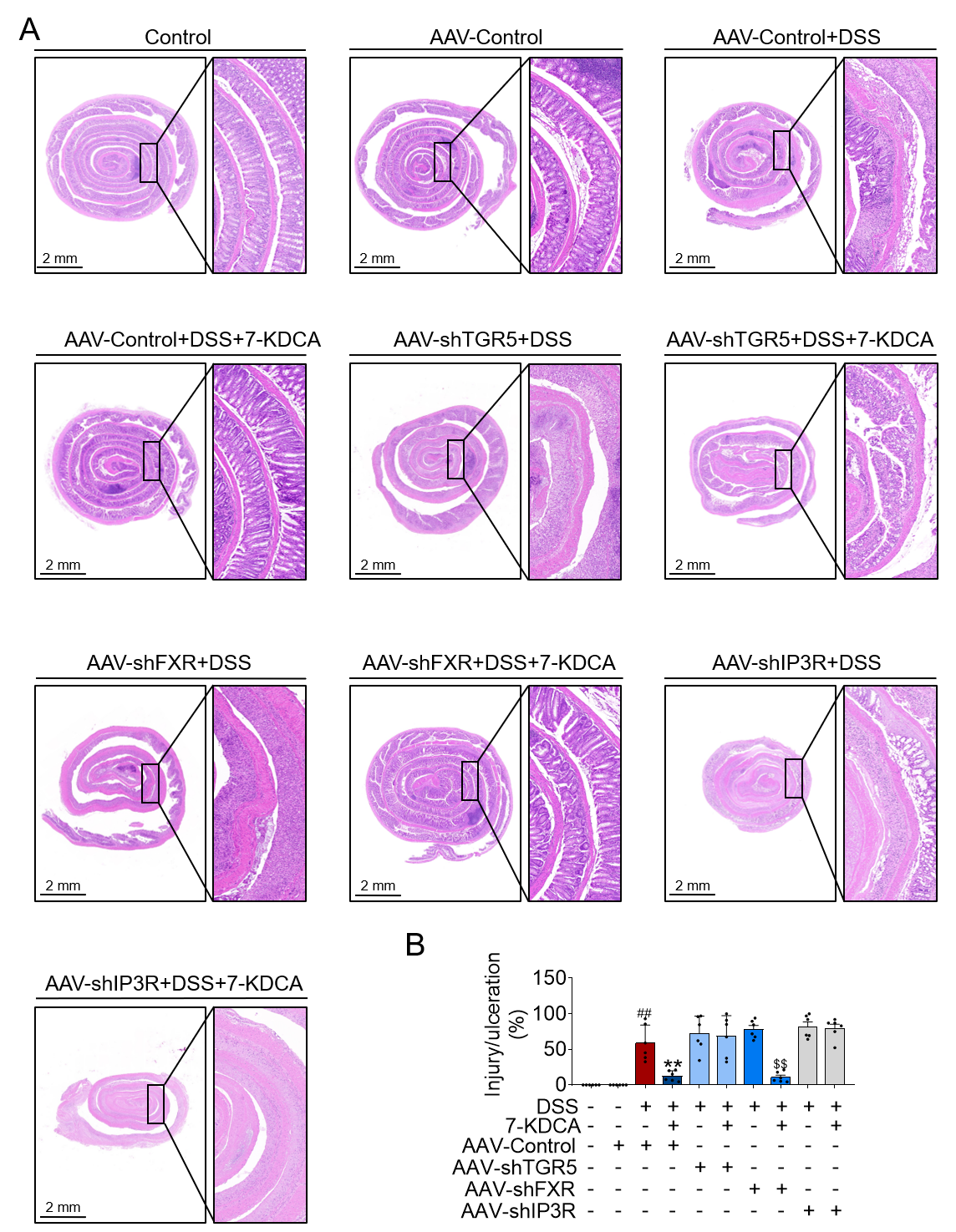
**

**Figure S27.** 7-KDCA facilitates colonic mucosal wound healing in FXR knockdown mice, while 7-KDCA fails to promotes colonic mucosal wound healing in colitis mice lacking TGR5 or IP3R. (A) Representative images of Swiss roll colon sections stained with H&E. The amplified details correspond to the black regions within the insets. (B) Histological colitis score denotes a ratio of the length of injured/ulcerated areas in relation to the overall length of the colon. This evaluation is conducted through Swiss roll mounts encompassing the entire colon. Data represent mean ± SD (n = 6). ^##^P < 0.01 vs. AAV-Control group. **P < 0.01 vs. DSS group. ^$$^P < 0.01 vs. AAV-shFXR group. 7-KDCA, 7-ketodeoxycholic acid; FXR, farnesoid X receptor; TGR5, Takeda G protein-coupled receptor 5; IP3R, inositol 1,4,5-triphosphate receptor; H&E, hematoxylin and eosin; DSS, dextran sulfate sodium.

**
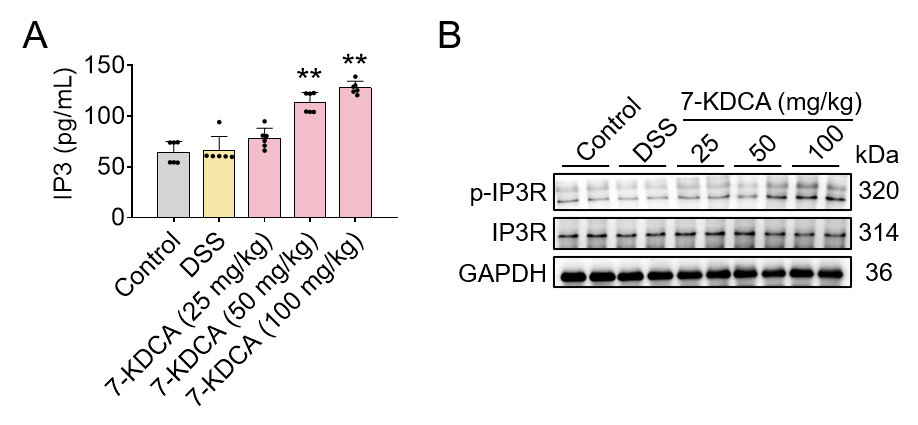
**

**Figure S28.** 7-KDCA activates IP3R in colon tissues in mice with DSS-induced colitis. (A) IP3 level in colon tissues was measured by ELISA. (B) Western blot analysis of IP3R and p-IP3R protein levels in colon tissues. Data represent mean ± SD (n = 6). **P < 0.01 vs. DSS group. 7-KDCA, 7-ketodeoxycholic acid; IP3, inositol 1,4,5-trisphosphate; IP3R, IP3 receptor; p-IP3R, phospho-IP3R; ELISA, enzyme-linked immunosorbent assay.


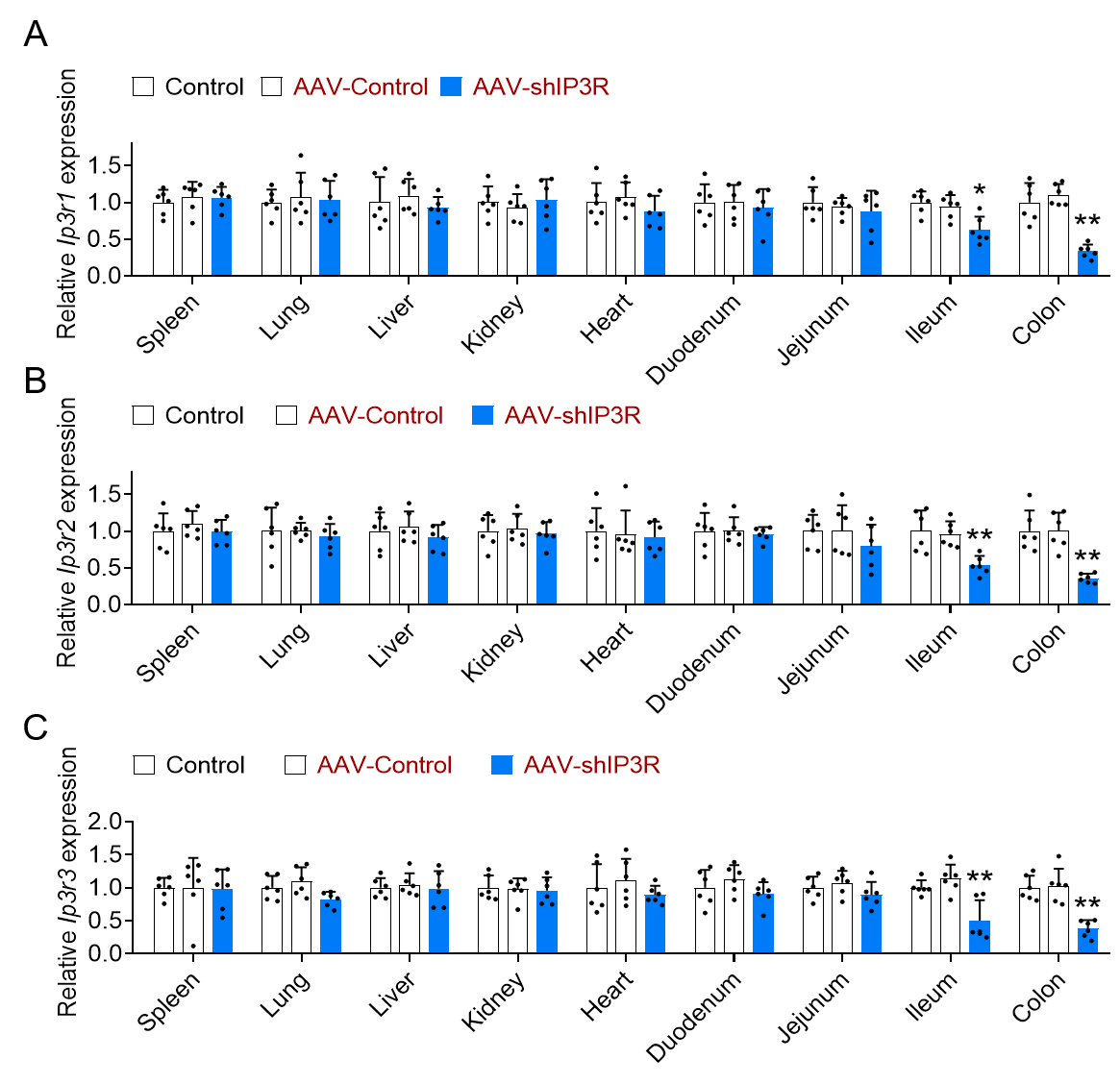


**Figure S29.** *Ip3r* expression was significantly blunted in ileum and colon from IP3R knockdown mice. The mRNA expression of *Ip3r1* (A), *Ip3r2* (B) and *Ip3r3* (C) in the spleen, lungs, liver, kidneys, heart, duodenum, jejunum, ileum and colon of mice by AAV-TGR5-shRNA enema. Data represent mean ± SD (n = 6). **P < 0.01 vs. AAV-Control group. IP3R, inositol 1,4,5-trisphosphate receptor.

**
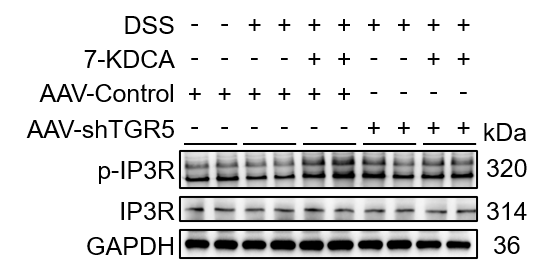
**

**Figure S30.** 7-KDCA is unable to activate IP3R in TGR5 knockdown mice. Western blot analysis of IP3R and p-IP3R protein levels in colon tissues. Data represent mean ± SD (n = 6). 7-KDCA, 7-ketodeoxycholic acid; IP3R, inositol 1,4,5-trisphosphate receptor; TGR5, Takeda G protein-coupled receptor 5; DSS, dextran sulfate sodium.

**
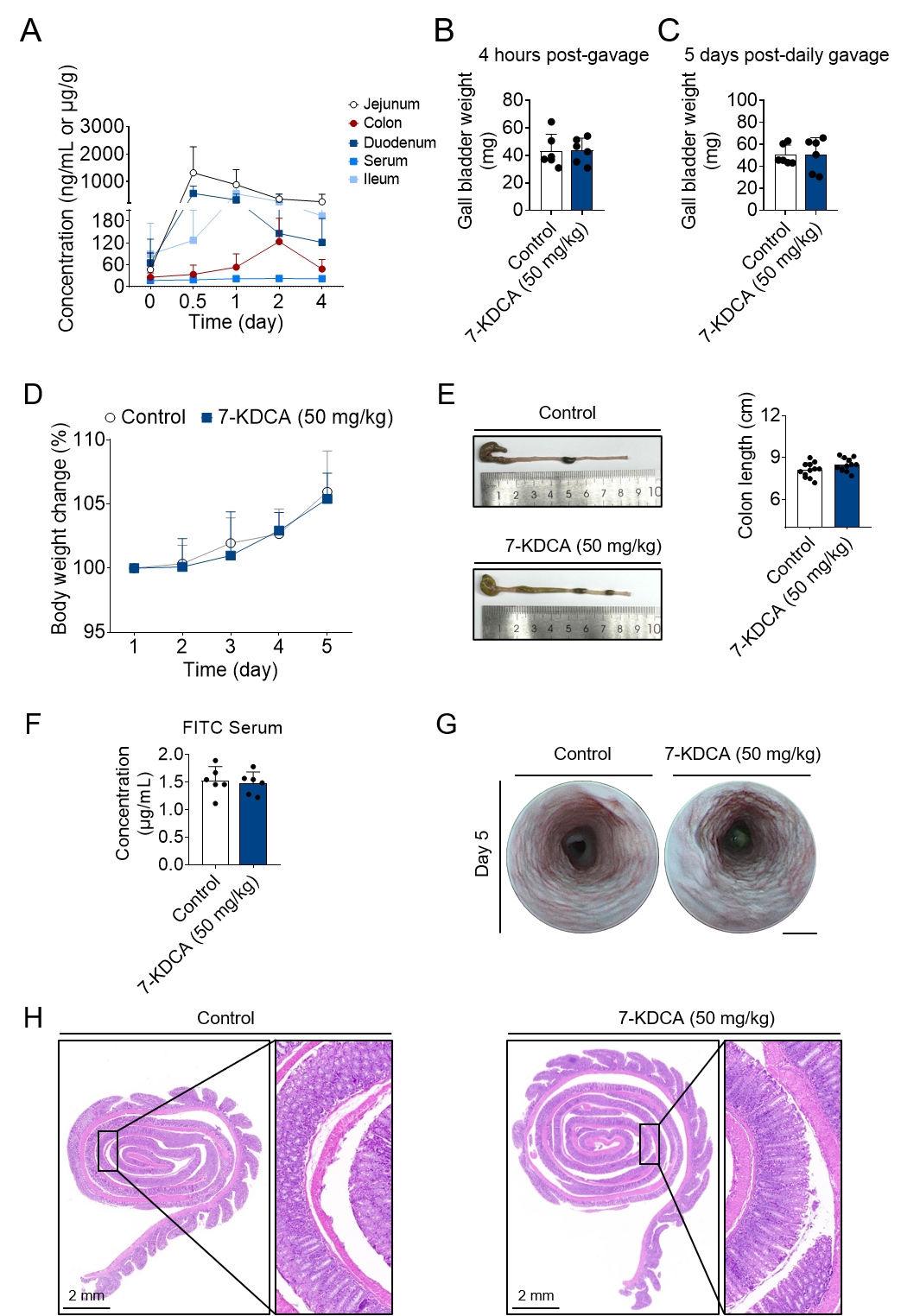
**

**Figure S31.** 7-KDCA itself does not disrupt intestinal function in healthy mice. C57/BL6J mice were treated with or without 7-KDCA for 5 days. (A) Concentrations of 7-KDCA in serum, duodenum, jejunum, ileum and colon. (B) Gallbladder weights were measured 5 h post-gavage with 7-KDCA. (C) Gallbladder weights were measured after 5 consecutive days of 7-KDCA gavage. (D) Percentage change in body weight. (E) Colon length. (F) Relative fluorescence intensity of FITC-dextran in serum. (G) Representative colonoscopy images of the colon. (H) Representative images of Swiss roll colon sections stained with H&E. The amplified details correspond to the black regions within the insets. Data represent mean ± SD (n = 6). 7-KDCA, 7-ketodeoxycholic acid; FITC, fluorescein isothiocyanate; H&E, hematoxylin and eosin.

**Table S1.** Identification of significantly differential metabolites in the sera of patients with ulcerative colitis based on UPLC-QTOF-MS/MS.

| Metabolites | Formula | Mass | Adjusted P-value | VIP | Fold change | UC & HC |
| --- | --- | --- | --- | --- | --- | --- |
| 7-Ketodeoxycholic acid | C_24_H_38_O_5_ | 406.272 | 0.0012188 | 2.167 | 0.40185 | ↓＊＊＊ |
| Linolenic acid | C_18_H_30_O_2_ | 278.225 | 0.00053983 | 1.978 | 0.38212 | ↓＊＊＊ |
| Indole-3-carboxaldehyde | C_9_H_7_NO | 145.158 | 0.0030317 | 1.000 | 0.76031 | ↓＊＊＊ |
| LysoPE (16:0) | C_21_H_44_NO_7_P | 453.559 | 0.0035405 | 1.076 | 1.4776 | ↑＊＊＊ |
| LysoPC (18:1) | C_26_H_52_NO_7_P | 521.678 | 0.0043788 | 1.284 | 1.4991 | ↑＊＊＊ |
| LysoPC (15:1) | [C_23_H_46_NO_7_P](https://lipidmaps.org/tools/ms/iso2d_Ag.php?formula=C23H46NO7P) | 479.301 | 0.0080719 | 1.119 | 1.5081 | ↑＊＊ |
| LysoPC (22:6) | [C_30_H_50_NO_7_P](https://lipidmaps.org/tools/ms/iso2d_Ag.php?formula=C30H50NO7P) | 567.333 | 0.010969 | 1.190 | 1.4937 | ↑＊＊ |
| LysoPE (18:1) | C_23_H_46_NO_7_P | 479.597 | 0.012116 | 1.169 | 2.0858 | ↑＊＊ |
| Glycohyocholic acid | C_26_H_43_NO_6_ | 465.309 | 0.016633 | 1.476 | 0.56924 | ↓＊＊ |
| Octanoyl-L-Carnitine | C_15_H_29_NO_4_ | 287.210 | 0.017495 | 1.381 | 0.59131 | ↓＊＊ |
| LysoPC (18:0) | C_26_H_54_NO_7_P | 523.364 | 0.023108 | 1.060 | 1.3888 | ↑＊＊ |
| Perfluorooctanoic acid | C_8_HF_15_O_2_ | 413.974 | 0.023249 | 1.311 | 0.68441 | ↓＊＊ |
| Indoleacetic acid | C_10_H_9_NO_2_ | 175.064 | 0.02498 | 1.039 | 0.72289 | ↓＊＊ |
| Panthenol | C_9_H_19_NO_4_ | 205.131 | 0.030484 | 1.727 | 1.7805 | ↑＊＊ |
| Pyroglutamic acid | C_5_H_7_NO_3_ | 129.115 | 0.030484 | 1.071 | 1.6146 | ↑＊＊ |
| LysoPC (20:1) | [C_28_H_56_NO_7_P](https://lipidmaps.org/tools/ms/iso2d_Ag.php?formula=C28H56NO7P) | 549.379 | 0.041088 | 1.203 | 1.9979 | ↑＊ |
| Allopurinol | C_5_H_4_N_4_O | 136.039 | 0.045562 | 1.507 | 2.2293 | ↑＊ |

The levels of differentially expressed metabolites were labeled with (↑) up-regulation and (↓) down-regulation (*, *P* < 0.05; **, *P* < 0.01; ***, *P* < 0.001).**Table S2.** Information of healthy volunteers and UC patients recruited by Jiangsu Province Hospital of Chinese Medicine.

| **Groups** | **Gender** | **Age（years）** | **Number** |
| --- | --- | --- | --- |
| Healthy Volunteers | Female | 40.67 ± 25.33 | 7 |
| UC patients | Male  Female | 37.25 ± 20.75  48.65 ± 31.35 | 7  14 |
|  | Male | 47.14 ± 22.86 | 18 |

**Table S3.** Nucleotide sequences of gene-specific primers employed for qRT-PCR, related to the experimental procedures.

| **Specifies** | **Gene name** | **Primer** | **Sequence of forward and reverse primers (5’ to 3’)** |
| --- | --- | --- | --- |
| ***Mus musculus*** | *Tnf-α* | Forward | GCCTCTTCTCATTCCTGCTTGTGG |
|  |  | Reverse | GTGGTTTGTGAGTGTGAGGGTCTG |
|  | *Il-6* | Forward | TCGCAGCAGCACATCAACAAGAG |
|  |  | Reverse | AGGTCCACGGGAAAGACACAGG |
|  | *Il-1β* | Forward | ACCTTCCAGGATGAGGACATGA |
|  |  | Reverse | AACGTCACACACCAGCAGGTTA |
|  | *Occludin* | Forward | TCGCCATATTTGCCTGTGTG |
|  |  | Reverse | CCAAAGAGCCCTGTCCCATA |
|  | *Tff3* | Forward | CTGCAGGAGACAGAATGCAC |
|  |  | Reverse | CTTGTGTTGGCTGTGAGGTC |
|  | *Tgf-β* | Forward | CCAGATCCTGTCCAAACTAAGG |
|  |  | Reverse | CTCTTTAGCATAGTAGTCCGCT |
|  | *Tgr5* | Forward | ACTGGTCCTGCCTCCTTCTCC |
|  |  | Reverse | ACACTGCCATGTAGCGTTCCC |
|  | *Fxr* | Forward | AAGTGTAAGAACGGGGGCAA |
|  |  | Reverse | TTCAGTTAACAAACATTCAGCCA |
|  | *Shp* | Forward | GTCCGACTAGTCTGTATGCACT |
|  |  | Reverse | CTACTGTCTTGGCTAGGACATC |
|  | *Fgf15* | Forward | CTCCAACTGCTTCCTCCGAA |
|  |  | Reverse | AGCCCGTATATCTTGCCGTG |
|  | *Ip3r1* | Forward | CTATGTGGATACGGAGGTGGAGATG |
|  |  | Reverse | GTGCTTCCTGTCGCTTGTGTTG |
|  | *Ip3r2* | Forward | GATGAGCAAGGAAGCAAGGTGAAC |
|  |  | Reverse | GAGTGAGATGTGCCTGGAGAACC |
|  | *Ip3r3* | Forward | TCGTCGGCTTCCTCTTCCTCAAG |
|  |  | Reverse | GCTGGCTGTGGAGTGGTTGTTG |
|  | *T-bet* | Forward | GGACCCAACTGTCAACTGCT |
|  |  | Reverse | AACTGTGTTCCCGAGGTGTC |
|  | *IFN-γ* | Forward | CTCTGAGACAATGAACGCTAC |
|  |  | Reverse | CTTCCACATCTATGCCACT |
|  | *GATA3* | Forward | ACCACCTATCCGCCCTATGT |
|  |  | Reverse | ACTCCCTGCCTTCTGTGCT |
|  | *IL-4* | Forward | CATCCTGCTCTTCTTTCTC |
|  |  | Reverse | CTTCTCCTGTGACCTCGTT |
|  | *RORγt* | Forward | CCCTCTGGCACACAATCTCT |
|  |  | Reverse | CGGTCCTCTGCTTCTCTTAGG |
|  | *IL-17* | Forward | GACTCTCCACCGCAATGAA |
|  |  | Reverse | GACCAGGATCTCTTGCTGGA |
|  | *Foxp3* | Forward | GGTACACCCAGGAAAGACAG |
|  |  | Reverse | ATCCAGGAGATGATCTGCTTG |
|  | *IL-10* | Forward | GCTATGTTGCCTGCTCTTACTG |
|  |  | Reverse | TCTGGCTGACTGGGAAGTG |
|  | *GAPDH* | Forward  Reverse | GGTTGTCTCCTGCGACTTCA  TGGTCCAGGGTTTCTTACTCC |
| ***Homo sapiens*** | *TGR5* | Forward | CCTAGGAAGTGCCAGTGCAG |
|  |  | Reverse | CTTGGGTGGTAGGCAATGCT |
|  | *FXR* | Forward | ACACTTTGCCTGTCTCCTGG |
|  |  | Reverse | AGACCCCTCCCCTGTAATCC |
|  | *SHP* | Forward | TCAAGTCCATTCCGACCAGC |
|  |  | Reverse | AAGAAGGCCAGCGATGTCAA |
|  | *FGF19* | Forward | AGATCAAGGCAGTCGCTCTG |
|  |  | Reverse | CGGATCTCCTCCTCGAAAGC |
|  | *CAR* | Forward | CCTCTTCTCTCCTGCTCCCT |
|  |  | Reverse | TCCCAGCATTTTCCCACTCC |
|  | *VDR* | Forward | TGGAGACTTTGACCGGAACG |
|  |  | Reverse | TCCCAGCATTTTCCCACTCC |
|  | *PXR* | Forward | AGTGAACGGACAGGGACTCA |
|  |  | Reverse | GAGACTCTGGCAACTCGCAG |
|  | *PRKACA* | Forward | GGATTGCCATCTACCAGAGGAAGG |
|  |  | Reverse | CTCCTTGCCACACTTCTCATTGATG |
